# Machine Learning Methodologies for Prediction of Rhythm-Control Strategy in Patients Diagnosed With Atrial Fibrillation: Observational, Retrospective, Case-Control Study

**DOI:** 10.2196/29225

**Published:** 2021-12-06

**Authors:** Rachel S Kim, Steven Simon, Brett Powers, Amneet Sandhu, Jose Sanchez, Ryan T Borne, Alexis Tumolo, Matthew Zipse, J Jason West, Ryan Aleong, Wendy Tzou, Michael A Rosenberg

**Affiliations:** 1 Colorado Center for Personalized Medicine University of Colorado School of Medicine Aurora, CO United States; 2 Division of Cardiology University of Colorado School of Medicine Aurora, CO United States; 3 Clinical Cardiac Electrophysiology Section Division of Cardiology University of Colorado School of Medicine Aurora, CO United States

**Keywords:** atrial fibrillation, rhythm-control, machine learning, ablation, antiarrhythmia agents, data science, biostatistics, artificial intelligence

## Abstract

**Background:**

The identification of an appropriate rhythm management strategy for patients diagnosed with atrial fibrillation (AF) remains a major challenge for providers. Although clinical trials have identified subgroups of patients in whom a rate- or rhythm-control strategy might be indicated to improve outcomes, the wide range of presentations and risk factors among patients presenting with AF makes such approaches challenging. The strength of electronic health records is the ability to build in logic to guide management decisions, such that the system can automatically identify patients in whom a rhythm-control strategy is more likely and can promote efficient referrals to specialists. However, like any clinical decision support tool, there is a balance between interpretability and accurate prediction.

**Objective:**

This study aims to create an electronic health record–based prediction tool to guide patient referral to specialists for rhythm-control management by comparing different machine learning algorithms.

**Methods:**

We compared machine learning models of increasing complexity and used up to 50,845 variables to predict the rhythm-control strategy in 42,022 patients within the University of Colorado Health system at the time of AF diagnosis. Models were evaluated on the basis of their classification accuracy, defined by the F1 score and other metrics, and interpretability, captured by inspection of the relative importance of each predictor.

**Results:**

We found that age was by far the strongest single predictor of a rhythm-control strategy but that greater accuracy could be achieved with more complex models incorporating neural networks and more predictors for each participant. We determined that the impact of better prediction models was notable primarily in the rate of inappropriate referrals for rhythm-control, in which more complex models provided an average of 20% fewer inappropriate referrals than simpler, more interpretable models.

**Conclusions:**

We conclude that any health care system seeking to incorporate algorithms to guide rhythm management for patients with AF will need to address this trade-off between prediction accuracy and model interpretability.

## Introduction

### Atrial Fibrillation

Atrial fibrillation (AF) affects an estimated 2.3 million Americans, with projections to over 10 million by the year 2050 [1,2], at current estimated costs of over US $26 billion each year in total [3] or US $18,000-US $20,000 per patient [4]. According to an analysis of the MarketScan database, patients diagnosed with AF underwent a mean 11.25 (SD 7.51) outpatient office visits, mean 4.74 (SD 5.24) outpatient hospital visits, and mean 0.71 (SD 1.28) emergency department visits, and were hospitalized for a mean 1.59 (SD 3.39) days on average over a given 6-month period [5]. Although the only treatment that has consistently reduced mortality from AF is the use of oral anticoagulation agents to prevent thromboembolic stroke [6-19], patients with AF can still have acute coronary syndromes, heart failure, and cardiovascular death at a rate of approximately 5% per year [20-23], including 35%-50% with hospital admissions or death within 5 years, even in the presence of oral anticoagulation [24,25]. Furthermore, the use of anticoagulation has no direct impact on the symptoms a patient may experience from AF, on the effect AF may have on underlying cardiovascular physiology, or on the long-term outcomes of being in AF rather than sinus rhythm. As such, the treatment of AF beyond identification of individuals needing anticoagulation is generally directed toward one of two strategies: (1) a rate-control strategy, focused solely on reducing the rate of ventricular excitation without attempting to restore sinus rhythm, or (2) a rhythm-control strategy, in which the focus is on restoring sinus rhythm using direct electrical energy (cardioversion), antiarrhythmic medications [26,27], catheter ablation, or a combination of two or more of these approaches [6,26,28-30]. Although a rate-control strategy can typically be performed under the care of a primary care physician, application of a rhythm-control strategy generally requires input from a specialist in cardiology or cardiac electrophysiology. Given the complexity of the decision about when to pursue a rhythm- or rate-control strategy, patients in whom a rhythm-control strategy is unlikely may be reflexively referred to cardiology or cardiac electrophysiology; in contrast, patients in whom a rhythm strategy would be beneficial may not be referred to a specialist who could provide this service. A method to identify patients who are more or less likely to undergo a rhythm-control strategy upstream could thus provide an attractive resource to improve care efficiency.

### Use of Electronic Health Records

The expansion of electronic health records (EHRs) has created the opportunity to develop automated methods of prediction using machine learning. Although machine learning methods can provide superior predictability over standard methods in some cases, this improved accuracy often comes at the expense of using *black box* methods for prediction, in which it is not clear what specific information is being used by a given model to make predictions [31]. Within the space of clinical decision-making, such opacity can be a problem as it not only prevents users from gaining trust in the model but also provides little feedback in terms of how potential factors might be modified to change a decision. Our group has previously described the application of machine learning methods to EHRs for the prediction of incident AF and other outcomes [32,33].

In this study, we applied a step-by-step process to develop prediction models of increasing complexity using EHR data to predict whether a given patient is likely to have a rate- or rhythm-control strategy at the time of diagnosis of AF. We structured our analysis to examine and compare methods that offer a range of levels of model interpretability as well as prediction accuracy. In conclusion, we have provided a set of models that can be applied using EHR data at the point of care to guide referrals for AF management broadly within a health care system.

## Methods

### Study Population

The University of Colorado (UC) Health hospital system includes 3 large regional centers (north, central, and south) over the front range of Colorado. All UC Health hospitals share a single Epic instance, with backups and storage within Epic’s Cogito Suite of databases, including Chronicles (operational database), Clarity (relational database), and Caboodle (dimensional database). In 2016, the UC entered into a unique partnership with Google to allow data from Caboodle to be loaded and stored in a research-focused data warehouse called the Health Data Compass, located entirely on the Google Cloud Platform, which was used by our team for this study. The data set was obtained using Google Big Query applied to the EHR system to return patients who were seen for outpatient encounters between October 11, 2010, and October 26, 2020, and were between the age group of 18 and 100 years at the index encounter, defined as the first time that a diagnosis of AF was entered for an outpatient seen at a UC Health clinic (see Multimedia Appendix 1, Table S1, for AF diagnosis definitions). The full data set contained 42,022 participants and was split into a training set (31,517/42,022, 75%) and a testing set (10,505/42,022, 25%), with model development performed using the training set and model comparisons using the testing set. This protocol was approved by the UC Multiple Institutional Review Board (#20-2192) using deidentified and uniquely encoded data sets, with a waiver of informed consent.

### Clinical Predictors

Clinical predictors were grouped into two broad categories, which were defined as *big data predictors* and *known predictors*. *Big data predictors* included any diagnosis (International Classification of Disease [ICD]-9 or ICD-10) or procedure event for each patient before the index encounter, as well as race, ethnicity, and financial class. Any medication that was active and administered via the oral route at the index encounter was also included as a big data predictor. For each participant, an array was created for active medications, procedures, and diagnoses, followed by the use of a tokenizer (*Keras Tokenizer*) to create a one-hot encoded data set with each unique medication, procedure, and diagnosis assigned its own variable, resulting in a data set containing 50,845 variables. *Known predictors* were defined as any cardiac or metabolic diagnoses that have been identified as having a potential association with the risk of AF, including hypertension (ICD-9 401.X; ICD-10 I10.X) [6,21,34], obesity (ICD-9 278.X; ICD-10 E66.X) [34-37], diabetes mellitus (ICD-9 250.X; ICD-10 E11.X), coronary artery disease (ICD-9 414.X; ICD-10 I25.X), and heart failure (ICD-9 428.X; ICD-10 I50.X) [21,24,34,38,39], and mitral valve disease (ICD-9 424.X or 394.X, ICD-10 I34.X), as well as age and sex. Age was normalized (mean subtracted and divided by SD) for all analyses except for logistic regression models and decision trees (not including random forests [RFs]), which used the unnormalized age. This allowed for improved optimization of the models that used the normalized age and greater interpretability of the models that used the unnormalized age. Missing values were imputed using the median value (continuous variables) or mode (discrete variables). No participants were missing age or sex, and diagnoses were assumed to be absent if the value was unavailable.

### Outcome: AF Treatment Strategy

AF treatments were defined as any medication, including antiarrhythmic medications, external cardioversion, or AF ablation procedure that was ordered within 6 months after the index encounter (Multimedia Appendix 1, Table S2). We defined the order for any antiarrhythmic medication, ablation, or cardioversion procedure as a *rhythm-control* strategy and any nodal agent or absence of a treatment order as a *rate-control* strategy. Treatments were only assessed following the index encounter (ie, the first outpatient visit at which the diagnosis of AF was entered); we did not examine subsequent treatments or study visits beyond the first 6 months after the index encounter. In one subanalysis, we examined the first selected rhythm-control strategy after the AF diagnosis, grouped into one of the following categories: antiarrhythmic medication, external cardioversion, and ablation.

### Modeling Strategy

#### Model Development

As the total number of participants to whom a rhythm-control strategy was applied was relatively low (imbalanced data), we first compared four methods of resampling: synthetic minority oversampling technique (SMOTE) [40,41], random oversampling, random undersampling, and Tomek links undersampling [42], as well as the use of raw features. Resampling was performed only in the training set.

Model development proceeded from the most interpretable (logistic regression) to the most complex and opaque (combined methods incorporating neural networks in ensemble format). Originally, we planned to run all models on both groups of inputs, known and big data predictors. However, we found that only deep learning models provided predictive accuracy for big data predictors. Thus, we ran the non–deep learning models on the known predictors only (Multimedia Appendix 1, Table S7). For logistic regression, we used the training data set to develop binary logistic regression classifiers for models of rate- versus rhythm-control and multinomial logistic regression for models of the first AF treatment strategy among those identified as having a rhythm-control strategy. For RFs, extreme gradient boosting, K-nearest neighbors, and naïve Bayes classification, grid search for hyperparameter optimization was performed using five-fold cross-validation on the training set, with manual grid optimization to ensure that the grid contained the optimal hyperparameters (ie, if a hyperparameter value was identified on the upper end of the grid range, the grid was expanded to ensure that the overall optimal hyperparameter was not beyond the bounds of the grid space).

The approach to fitting neural networks was to first increase the complexity (lower learning rate and increased numbers of layers and neurons) to improve fit on the training data and then to include regularization methods (eg, decrease the learning rate and add dropout) as the out-of-sample loss began to increase, as noted in the examination of learning curves (Multimedia Appendix 1, Figure S1). We used feed-forward neural networks for deep learning architecture. Unless described otherwise, neural networks used fully connected layers with Elu activation (except the final layer, sigmoid), He initialization, L2 regularization (Penalty=0.01), dropout (20%), batch normalization, binary cross-entropy loss, Root Mean Square Propagation optimizer with learning rate=1e-4, ρ=0.9, and 50 training epochs with early stopping. Formal comparisons of predictive accuracy are presented; any model structure or hyperparameters that are not presented can be assumed to have provided inferior predictive accuracy compared with the presented models.

We also examined several ensemble methods by integrating the optimal model on the basis of big data predictors (from neural networks) with known predictors to allow interpretability of the impact of each component on the overall prediction accuracy. We first included the predicted probability of a rhythm-control strategy for each participant on the basis of the neural network as an input into either a RF or logistic regression, with SMOTE resampling for the training set. We also examined the weights and structure of the neural network with big data inputs combined with auxiliary input from known predictors concatenated at the final layer, followed by the addition of a fully connected layer (called *neural network combined*) with sigmoid output to predict rhythm-control strategy. Weights from pretrained layers of the former models were frozen, with training only on additional layers after the addition of known predictors.

#### Model Interpretation

Our main goal was to identify an optimal model to predict the probability of providers applying a rhythm-control strategy on the basis of classification accuracy and interpretability. Classification accuracy was defined primarily by the F1 score, with supportive metrics including the area under the receiver operator characteristic curve (AUC), precision (positive predictive value), recall (sensitivity), accuracy (% correct predictions), and inspection of the 2 × 2 contingency table. Interpretability was examined by inspecting the relative importance of each predictor according to the metrics available for each modeling approach. For logistic regression, importance was defined by the chi-square statistic from a nested likelihood ratio test, with and without inclusion of the predictor in the model. For RFs, importance was defined by the Gini index, which describes the mean decrease in impurity across all nodes, averaged over all decision trees [43]. We also examined individual decision trees manually for the interpretability and relevance of decision cut-points.

Model calibration was assessed using calibration curves created by binning the predicted probability from each model over the deciles of prediction and examining the actual proportion of rhythm-control strategies within each decile. Receiver operator characteristic and precision-recall curves were plotted using standard methods (*sklearn*). To allow inspection of these models within the context of triggering referrals for evaluation of the rhythm-control strategy, we also plotted the proportion of appropriate, inappropriate, and missed appropriate referrals according to varying probability thresholds from each prediction model. These classifications were assigned by comparing whether a rhythm-control strategy was predicted by the model and whether it was actually used for each participant. Thus, *appropriate referrals* indicated the participants for whom a rhythm-control strategy was predicted and used, *inappropriate referrals* indicated those for whom a rhythm-control strategy was predicted but not used (false positives), and *missed appropriate referrals* indicated those for whom a rhythm-control strategy was not predicted but was used (false negatives).

### Computing Resources

Analyses and marginal estimation using logistic regression applied to the known predictors were conducted using Stata, IC (version 16, StataCorp, Inc). Analyses using both known and big data predictors were performed using scripts written in Python 3.7.4, with dependencies (software packages) including the following: *imblearn 0.0, Keras 2.2.4, numpy 1.19.4, pandas 0.25.1, scikit-learn 0.23.2,* and *tensorflow 2.4.0.* Scripts were developed and tested using Jupyter Notebook and deployed using command line programming at the UC’s Health Data Compass Eureka virtual environment, hosted on Google Cloud Platform, using 64 central processing units and approximately 8-10 GB RAM, depending on the modeling requirements.

## Results

### Known Predictors

**Figure 1:**
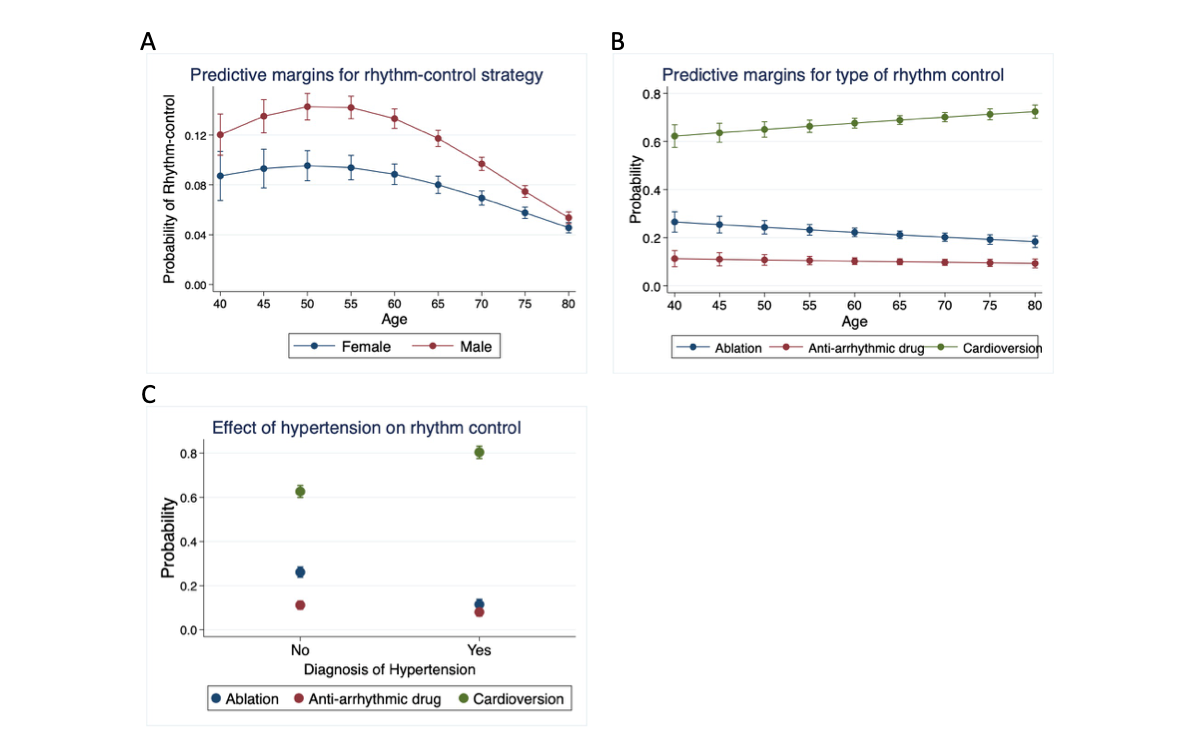
(A) Predictive margins for rhythm-control strategy. Based on logistic regression with age and age-squared and age-sex interactions. Error bars represent the 95% CIs applied to each age-sex combination. (B) Predictive margins for the type of rhythm-control strategy: ablation, antiarrhythmic drug, and external cardioversion. Based on multinomial logistic regression for the first rhythm-control treatment applied, with age and age-squared and age-sex interactions. Error bars represent the 95% CI applied to each age-sex combination. (C) Predictive margins for the effect of hypertension diagnosis on the rhythm-control strategy. Based on multinomial logistic regression for the first rhythm-control treatment applied, with age and age-squared and age-sex interactions. Error bars represent the 95% CI applied to each age-sex combination.

The overall study population demographics are provided in Table 1, split according to the strategy deployed (rate vs rhythm-control) and the training or testing set. A rhythm-control strategy was ordered within 6 months of AF diagnosis in 7.51% (3155/42,022) of patients. On average, patients undergoing a rhythm-control strategy were younger and male, with lower rates of existing cardiac conditions other than obesity. Among patients ordered for a rhythm-control strategy (and for whom this information was available), 20.88% (495/2370) were first ordered for ablation, 9.7% (230/2370) were ordered for an antiarrhythmic medication, and 69.41% (1645/2370) were ordered for external cardioversion. All known predictors (Table 1), except for obesity and hypertension, were significantly associated with a rhythm-control strategy at *P*<.005 (after Bonferroni adjustment for multiple comparisons). Nonlinearity of the interaction with age and sex was notable (Figure 1); younger men were more likely to have a rhythm-control strategy, with normalization of the sex-dependent effect by older age. Among the individuals in whom a rhythm-control strategy was ordered, the age-sex interaction remained significant, although the relationship between age and probability of rhythm-control strategy was no longer nonlinear (Figure 1). In addition, hypertension diagnosis was the strongest predictor of the type of rhythm-control strategy used. Individuals with a previous diagnosis of hypertension were less likely to have an ablation or antiarrhythmic medication and more likely to have a cardioversion ordered (Figure 1).

**Table 1:** Population demographics.

Demographics	Training set (n=31,517)			Testing set (n=10,505)	
	Rhythm control (n=2370)	Rate control (n=29,147)	Rhythm control (n=785)		Rate control (n=9720)
Age (years), mean (SD)	66.4 (12.0)	72.1 (12.9)	67.1 (11.6)		72.3 (12.7)
Sex (female), n (%)	779 (32.9)	12,588 (43.2)	265 (33.8)		4115 (42.3)
HTN^a^, n (%)^b^	1036 (43.7)	14,577 (50)	372 (47.4)		4870 (50.1)
Obesity, n (%)^c^	366 (15.4)	3877 (13.3)	156 (19.9)		1243 (12.8)
Diabetes, n (%)^d^	343 (14.5)	5305 (18.2)	115 (14.7)		1768 (18.2)
CAD^e^, n (%)^f^	475 (20)	7433 (24.5)	164 (20.9)		2497 (25.7)
Heart failure, n (%)^g^	488 (20.6)	5625 (19.3)	142 (18.1)		1874 (19.3)
Mitral valve disease, n (%)^h^	394 (16.6)	4841 (16.6)	124 (15.8)		1687 (17.4)

^a^HTN: hypertension diagnosis.

^b^International Classification of Disease-9 401.X; International Classification of Disease-10 I10.X.

^c^Obesity diagnosis (International Classification of Disease-9 278.X; International Classification of Disease-10 E66.X).

^d^Diabetes mellitus (International Classification of Disease-9 250.X; International Classification of Disease-10 E11.X).

^e^CAD: coronary artery disease.

^f^International Classification of Disease-9 414.X; International Classification of Disease-10 I25.X.

^g^Heart failure (International Classification of Disease-9 428.X; International Classification of Disease-10 I50.X).

^h^Mitral valve disease (International Classification of Disease-9 424.X or 394.X; International Classification of Disease-10 I34.X).

**Figure 2:**
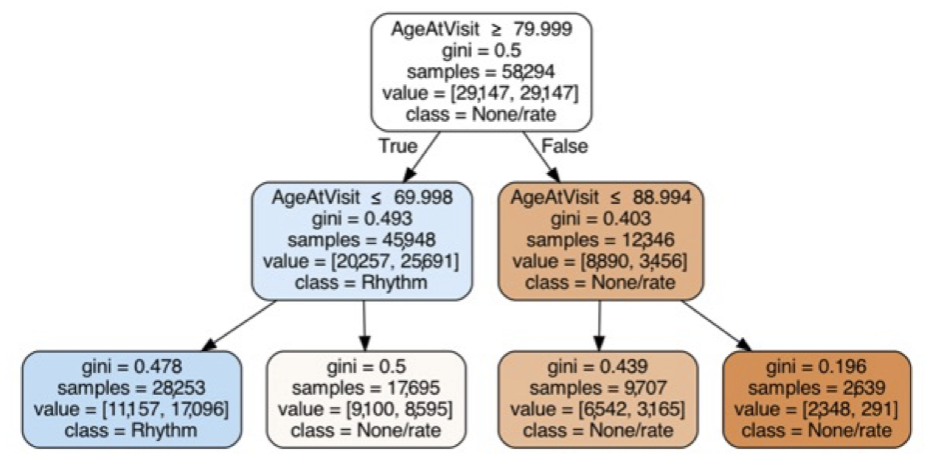
Decision tree for rhythm-control strategy. Based on known predictors to classify rate- versus rhythm-control strategy using the training data. Maximum depth=2, minimum samples to split nodes=50.

Among the supervised learning algorithms to predict a rhythm-control strategy based only on known predictors (Multimedia Appendix 1, Table S3), we found that all methods had a similar magnitude of F1 score and that some resampling method (SMOTE being most common) was needed for optimal prediction (Table 2). Feature importance applied to the highest performing RF model demonstrated that age was by far the strongest predictor (Table 3). Inspection of the decision tree (Figure 2) indicated that age <70 years was strongly associated with a rhythm-control strategy, and age >89 years was strongly associated with the rate-control strategy. When the models were tested on age-stratified data, there was a slight improvement in the average AUC associated with increased age, but this was not statistically significant (Multimedia Appendix 1, Figure S3). The logistic regression results showed similar relative importance for the features, although RF favored coronary artery disease slightly more than sex as a predictor compared with the logistic regression, and mitral valve disease was relatively less important for regression than RF (Table 3).

**Table 2:** Best supervised learning models.

Model^a^	Resampling	F1 score	AUC^b^	Accuracy	Recall	Precision
Random forest^c^	SMOTE^d^	0.186	0.591	0.689	0.476	0.116
Extreme gradient boosting^e^	Random oversampling	0.179	0.591	0.614	0.563	0.106
K-nearest neighbors^f^	Random undersampling	0.181	0.605	0.541	0.682	0.105
Naïve Bayes^g^	SMOTE	0.184	0.602	0.596	0.609	0.108
Logistic regression	SMOTE	0.185	0.608	0.570	0.654	0.108

**^a^**All models except neural network applied to known predictors only.

^b^AUC: area under the receiver operator characteristic curve.

^c^Random forest hyperparameters: estimators=200, maximum features=8, maximum leaf nodes=300.

^d^SMOTE: synthetic minority oversampling technique.

^e^Extreme gradient boosting hyperparameters: booster=gbtree, η=0.9, γ=0, α=1, λ=0.

^f^K-nearest neighbors: N=500.

^g^Naïve Bayes: α=0.

**Table 3:** Feature importance.

Predictor	Random forest impurity reduction^a^ (%)	Logistic chi-square (*df*)	*P* value
Age (years)	81.74	462.11 (4)	<.001
CAD^b^	3.25	21.28 (1)	<.001
Sex	3.01	60.61 (3)	<.001
Mitral valve disease	2.82	8.04 (1)	.01
Diabetes mellitus	2.78	18.46 (1)	<.001
Heart failure	2.43	17.59 (1)	<.001
Hypertension	2.36	4.03 (1)	.04
Obesity	1.62	2.61 (1)	.11

^a^For random forest (synthetic minority oversampling technique resampling).

^b^CAD: coronary artery disease.

### Big Data Predictors

**Figure 3:**
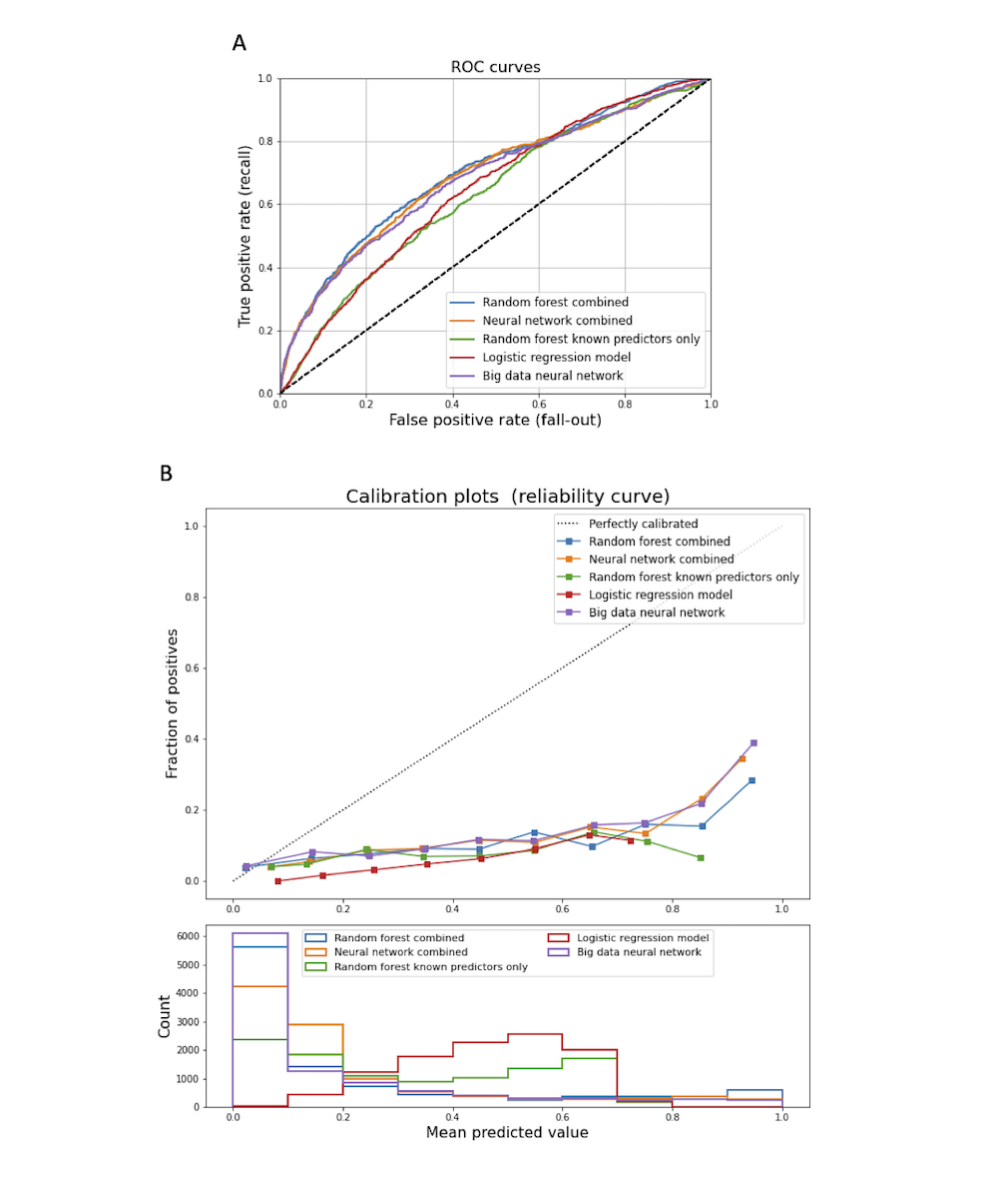
(A) Receiver operator characteristic curves for prediction models. Shown are top five models, including random forest combined and neural network combined (use big data and known inputs), random forest and logistic regression (use only known inputs), and neural network (only big data inputs). (B) Calibration curves (top) and histograms (bottom) for prediction models. Shown are top five models, including random forest combined and neural network combined (use big data and known inputs), random forest and logistic regression (use only known inputs), and neural network (only big data inputs). ROC: Receiver operator characteristic.

For big data predictors, only neural networks provided an F1 score over 0.0, so we focused on identifying the optimal neural network to predict a rhythm-control strategy. Across all neural networks using raw features, SMOTE, or random undersampling, we found that a 2-layer neural network with SMOTE provided superior prediction accuracy on the basis of the F1 score (Multimedia Appendix 1, Table S4). When examined within the context of logistic regression, decision tree, and RF, predictions from the big data neural network were by far the most predictive (Multimedia Appendix 1, Table S5). When combined as an ensemble with RF (RF combined) and neural network (neural network combined), the predictive accuracy remained high, with comparable F1 scores across models (Table 4) and clear improvement in prediction compared with RF or logistic regression based only on known predictors (Figure 3). Examination of calibration (Figure 3) indicated that all models were poorly calibrated and tended to overfit the data (predict rhythm-control strategy more often than this strategy was ordered).

**Table 4:** Combined big data (BD) and known predictor models.

Model	F1 score	AUC^a^	Accuracy	Recall	Precision
Random forests combined	0.258	0.643	0.807	0.451	0.181
Neural network combined	0.250	0.617	0.843	0.350	0.194
Neural network (BD predictors)	0.260	0.629	0.835	0.387	0.195

^a^AUC: area under the receiver operator characteristic curve.

**Figure 4:**
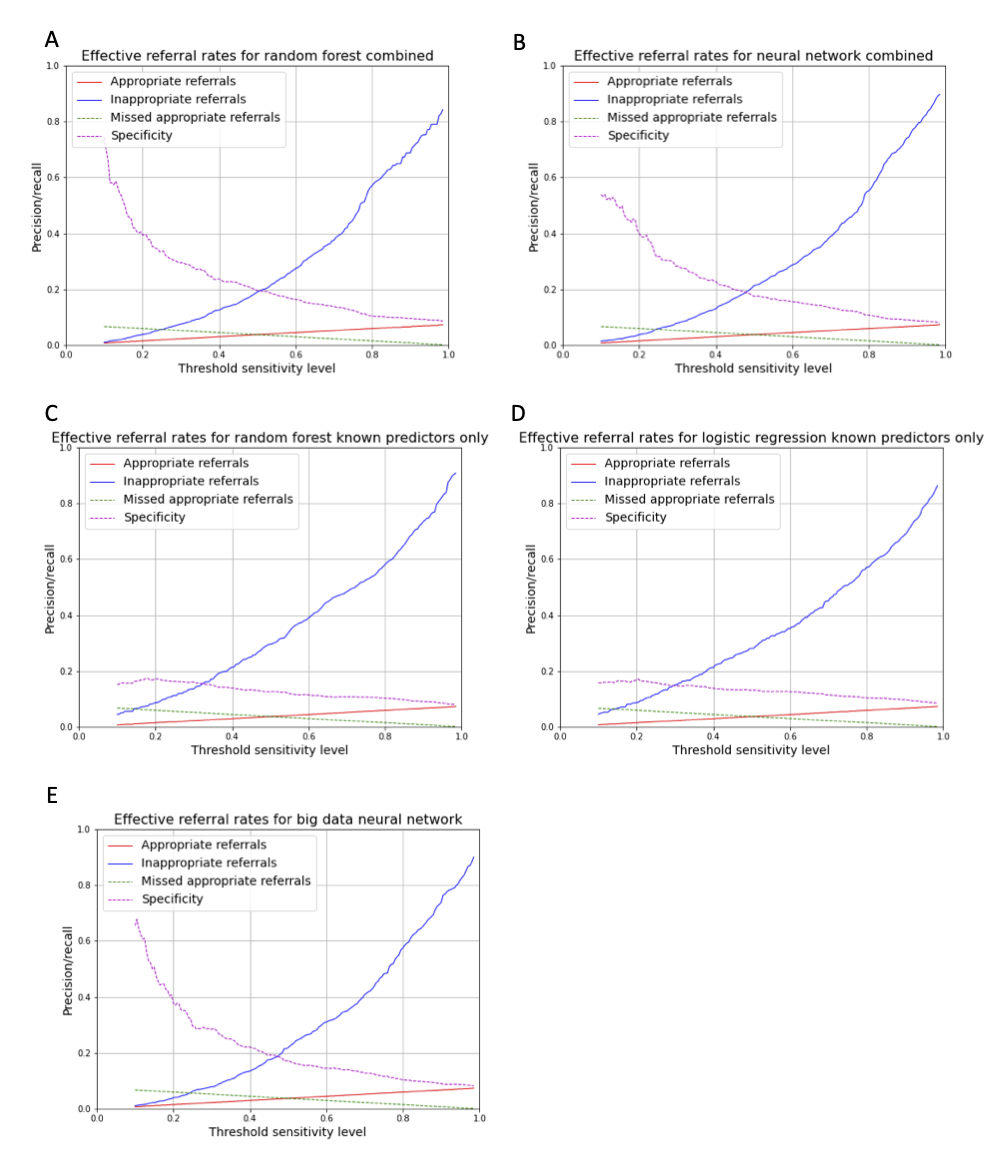
Decision curves for prediction models based on proportion of appropriate and inappropriate referrals that would result from applying the model at different levels of sensitivity (thresholds): (A) random forest combined, (B) neural network combined, (C) random forest, (D) logistic regression, and (E) neural network.

On the basis of precision-recall analysis (Multimedia Appendix 1, Figure S2), we examined the rate of appropriate, inappropriate, and missed appropriate referrals that would result from implementing an automated algorithm using these models at the time of AF diagnosis (Figure 4; Multimedia Appendix 1, Table S6). As expected, we found that the proportion of appropriate referrals (referral when rhythm-control strategy is likely) increased and missed appropriate referrals decreased with an increase in the sensitivity (recall) threshold used to guide the decision. However, it was also found that more complex models, such as those using combined known and big data predictors within a black box context, had a lower rate of inappropriate referrals for thresholds between 0.3 and 0.8. To put this in context, if the model was applied to 10,000 patients at the time of AF diagnosis, increasing the sensitivity (recall) threshold from 0.5 to 0.7 would decrease the number of missed appropriate referrals by 150 patients for both models, at the expense of an increase in the number of inappropriate referrals of 1690 (logistic regression) to 1850 (RF combined). The use of models based solely on known predictors would increase the proportion of inappropriate referrals by approximately 20% compared with those that included big data predictors (Figure 4; Multimedia Appendix 1, Table S6).

## Discussion

### Principal Findings

In this EHR-based observational study of automated algorithms for the prediction of a rhythm-control strategy, we made several observations about the modeling process and the impact of using greater amounts of data to guide referrals. First, we found that nearly all methods were significantly improved by integration of some form of resampling during training (SMOTE being the most effective generally), which has been described previously by our group and others for the prediction of imbalanced outcomes. Although these approaches tended to improve the prediction accuracy as assessed by the F1 score and other measures of classification, they resulted in models that tended to predict a rhythm-control strategy more often than one was actually used, suggesting that they were overfitting the data. This result is consistent with previous work using machine learning to predict rare outcomes from EHR data by our team, including the prediction of AF itself [33] and myocardial infarction [32].

Second, we found that only neural networks could provide the computational power to produce accurate prediction models with big data inputs; none of the other approaches provided an AUC over 0.5 (F1 score>0.0) when applied to big data inputs. This result is also similar to previous findings with the application of machine learning to EHR data [32,33] and suggests the power of deep learning over standard methods, which has been demonstrated widely across a range of applications [44-46].

Finally, and most interestingly, we found that although no method was clearly superior to the others, there appeared to be a trade-off in which more interpretable models on the basis of known predictors alone provided inferior predictive accuracy compared with the use of more opaque, black box approaches incorporating deep neural networks. Specifically, we found that a model based solely on age could be reasonably effective for identifying patients in whom a rhythm-control strategy could be applied, but that greater levels of predictive accuracy required incorporation of much larger amounts of information, at the expense of not knowing which specific predictors (diagnoses, medications, or prior procedures) among the over 50,000 were needed. The benefit of using these more complex models was evident in a lower rate of inappropriate referrals within a wider range of thresholds, in which increasing the sensitivity of the predictions to decrease the number of missed appropriate referrals resulted in approximately 20% more inappropriate referrals for all but the lowest and highest thresholds. The bottom line is that a health system seeking to implement a clinical decision support algorithm could find a substantial increase in the costs due to inappropriate referrals in order to apply a more interpretable approach to guiding clinical decisions.

This study offers several comments, and the broader implications applied to both decisions about rhythm-control strategies and the role of machine learning and statistical modeling in EHR-based clinical decision support. In terms of rhythm- versus rate-control strategies, there are little data about the best approach for a given patient at the time of AF diagnosis. Early clinical trials limited to antiarrhythmic medications for rhythm-control showed no difference in outcomes for rhythm-control compared with a rate-control strategy [47-50], although more recent trials that include AF ablation for rhythm-control have noted improvements in ventricular function [21,38] and lower rates of stroke and death among patients with heart failure treated using a rhythm-control strategy that included AF ablation [24,51-53]. The recently published Early Treatment of Atrial Fibrillation for Stroke Prevention Trial 4 [54] examined early application of a rhythm-control strategy (within a year) and noted a reduction in the combined outcome of cardiovascular death, stroke, or cardiac hospitalizations [48,50,55], although the study did not directly measure costs [55]. Within the context of an automated referral algorithm, increasing the number of referrals blindly across the population is unlikely to be cost-effective, as we found that there was overall a relatively low rate (3155/42,022, 7.51%) of patients who had a rhythm-control strategy ordered within a 6-month period. In contrast, a program that avoids referrals for rhythm-control due to the overall low rate is likely to result in many patients being denied the opportunity to undergo treatment that could improve morbidity and mortality. We did not specifically examine long-term outcomes in this investigation, although we anticipate that like many models of automated decision-making, the procedure must start by mimicking expert decisions before moving on to models that incorporate outcomes. For example, the AlphaGo computer algorithm for playing Go began with modeling expert moves in the first version [56] before using automated game simulation to identify a model that could achieve suprahuman performance [57].

With regard to the use of deep learning models to make predictions about clinical decisions, there is an important issue of out-of-sample predictive accuracy, which includes model overfitting—fitting noise in the training data set that results in reduced predictive accuracy in the testing and validation data set—as well as sampling bias related to the population used to derive the prediction model being different from that in which it is applied. One of the remarkable features of modern deep learning methods is that through regularization techniques, such as dropout, these models are capable of fitting data in which the number of trainable parameters is greater than the number of samples or participants. However, due to the *curse of dimensionality*, the use of such a large number of predictors results in a large space of extrapolation (few data points *nearby* one another), in such a manner that only through trial and error, and use of strictly held-out testing data sets, can one increase the probability of fitting signal rather than noise. Even with careful attention to learning curves, one still cannot be certain of a model’s predictive robustness without continued validation in external data sets. Such work is planned for these models, in which the trade-off between the use of a simple model with highly mappable inputs but lower predictive accuracy is balanced against the use of a complex deep learning model with greater accuracy; however, this requires a method to directly map approximately 50,000 features to the model input for application. Ultimately, more work will be needed to understand both the conceptual challenges of deep learning for clinical decision-making related to bias and overfitting, as well as the practical issues of how one applies a model developed in one EHR to another.

### Limitations

As expected from the examination of clinical decision-making using EHR data alone, there are several limitations to our study. First, as a result of the sheer number of encounters analyzed, we were unable to provide a manual chart or clinical validation of the decisions made in terms of rate or rhythm-control. As we defined the first diagnosis of AF as the first time it was entered into the EHR, it is highly likely that participants may have had undocumented AF before the index encounter and that a rate- or rhythm-control strategy may have been addressed at that point in time or by providers outside of our health care system. In addition, it is possible that many AF diagnoses were made in error and that patients may have had atrial flutter or supraventricular tachycardia rather than AF, in which case rate versus rhythm-control decisions would be irrelevant. Although we have an ongoing project to examine decisions at a patient-by-patient level, such an approach would not scale for the purposes of this analysis. Second, we selected an arbitrary 6-month window over which to assign a patient to a given strategy on the basis of whether a known rhythm-control approach was ordered. We were thus blind to patients who might have undergone a rhythm-control strategy outside the 6-month window or patients who started out with a rhythm-control strategy but then changed to rate-control going forward. Finally, although we were able to collect EHR-based data to apply predictive models, we were unable to obtain perhaps more relevant data pertaining to the decision about rate or rhythm control as it is applied clinically, such as symptoms or patterns of AF presentation. Clinically, symptoms are among the strongest reasons for referral for evaluation of AF by experts, and the inability to measure the symptoms with which a patient presents and how they progress is a limitation of our approach. Additional work using natural language processing of clinical notes or integration of other types of data related to patient activity or symptoms could provide a solution, although such data were not available at the time of this analysis. Importantly, the combined methodology we have described could be easily expanded to include this information without the need to retrain models entirely and could be directly analyzed in the same manner in which we integrated known predictors of AF alongside 50,000 big data inputs for prediction.

### Conclusions

Historically, the direct application of clinical decision models was limited by data input capacity, integration of analytics with data storage, and the inability to deliver results directly at the point of care. However, advances in computer technology over the past 30 years have provided solutions to these problems toward the goal of incorporating artificial intelligence into clinical decision-making. The recent expansion of EHR use now provides vast amounts of data that can be collected, stored, and applied for clinical prediction at the point of care, without the need for manual data entry. These advances have created the opportunity for fully integrated artificial intelligence–based decision analysis at a scale previously unseen in clinical investigations, as well as allowing for dynamic updating of prediction models over time as greater amounts of data are collected and technologies and treatment options expand. This study is among the first to apply machine learning within the clinical decision context using this massive amount of data in a manner that could be directly applied within a health care system. The trade-off between model interpretability and predictive accuracy that we found is likely to be repeated across many future applications in which understanding the role of predictors is balanced against thousands, and potentially millions, of dollars in unnecessary referrals if such a system were automated. Clearly, more work is required before these systems can be implemented without oversight from a clinician; however, as we have noted, administrators and health care decision-makers should be aware that there is likely to arise a situation in which interpretability comes with a cost.

## Appendix

Multimedia Appendix 1 Supplemental figures and tables.

## Abbreviations

AF: atrial fibrillation
AUC: area under the receiver operator characteristic curve
EHR: electronic health record
ICD: International Classification of Disease
RF: random forest
SMOTE: synthetic minority oversampling technique
UC: University of Colorado
