# Supplementary material for: Machine Learning Methodologies for Prediction of Rhythm-Control Strategy in Patients Diagnosed With Atrial Fibrillation: Observational, Retrospective, Case-Control Study
Source: JMIR Med Inform. 2021 Dec 6;9(12):e29225. doi: 10.2196/29225 (PMC8691402; doi:10.2196/29225)
Supplement: Multimedia Appendix 1 [file medinform_v9i12e29225_app1.pdf]

MULTIMEDIA APPENDIX

Table S1. Diagnosis codes for atrial fibrillation
Table S2A. Anti-arrhythmic medications
Table S2B. Nodel medications
Table S2C. Ablation procedure codes
Table S2D. Cardioversion procedure codes

Figure S1. Learning curve example for neural network training

Table S3A. Random forest comparison using known predictors
Table S3B. Extreme gradient boosting comparison using known predictors
Table S3C. K-nearest neighbors (KNN) comparison using known predictors.
Table S3D. Naïve Bayes comparison using known predictors.

Table S4A. Neural Network Training Results
Table S4B. 2x2 Contingency Tables for each model

Figure S2A-E. Precision-Recall Curves for Prediction Models. Random Forest Combined (Fig. S2A), Neural Network Combined (Fig. S2B), Random Forest (Fig. S2C), Logistic Regression (Fig. S2D), and Neural Network (Fig. 2E).

Table S5A. Odds ratios and statistics for regression model with Big Data Prediction
Table S5B. Feature Importance of Random forest model with Big Data Prediction
Table S5C. Confusion Matrices for each combined model

Tables S6A-6E. Values for proportions of graphs. Table S6A. Neural Network Combined; Table S6B. Random Forest Combined; Table S6C. Random Forest Known Predictors only; Table S6D. Logistic Regression Known Predictors only; Table S6E. Neural Network BD Predictors only

Figure S3. Select models stratified by age at time of visit

Table S7. All model, data, and hyperparameter permutations tested

Table S1. Atrial Fibrillation Diagnosis

concept_name	vocabulary_id	concept_class_id	concept_code
Unspecified atrial fibrillation and atrial flutter	ICD10CM	4-char nonbill code	I48.9
Atrial fibrillation	ICD9CM	5-dig billing code	427.31
Atrial fibrillation and atrial flutter, unspecified	ICD10	ICD10 code	I48.9
Unspecified atrial fibrillation	ICD10CM	5-char billing code	I48.91
Atrial fibrillation and flutter	ICD9CM	4-dig nonbill code	427.3
PAROXYSMAL ATRIAL FIBRILLATION	OXMIS	OXMIS	4279AP
Atrial fibrillation and flutter	Read	Read	G573.00
Permanent atrial fibrillation	Read	Read	G573400
Non-rheumatic atrial fibrillation	Read	Read	G573300
Atrial fibrillation	Read	Read	G573000
Atrial fibrillation resolved	Read	Read	212R.00
Chronic Atrial Fibrillation	CIEL	Diagnosis	151873
rapid atrial fibrillation	CIEL	Diagnosis	158626
controlled atrial fibrillation	CIEL	Diagnosis	155747
personal history of atrial fibrillation	CIEL	Diagnosis	158302
Lone Atrial Fibrillation	CIEL	Diagnosis	135636
Atrial Fibrillation and Flutter	CIEL	Diagnosis	121345
Paroxysmal Atrial Fibrillation	CIEL	Diagnosis	130790
Atrial fibrillation detected	SNOMED	Clinical Finding	1066831000000100
Pre-excited atrial fibrillation	SNOMED	Clinical Finding	762247006
Controlled atrial fibrillation	SNOMED	Clinical Finding	300996004
Atrial fibrillation with rapid ventricular response	SNOMED	Clinical Finding	120041000119109
Persistent atrial fibrillation	SNOMED	Clinical Finding	440059007
Lone atrial fibrillation	SNOMED	Clinical Finding	233910005
Atrial fibrillation	SNOMED	Clinical Finding	49436004
Non-rheumatic atrial fibrillation	SNOMED	Clinical Finding	233911009
ECG: atrial fibrillation	SNOMED	Clinical Finding	164889003
Atrial fibrillation resolved	SNOMED	Clinical Finding	196371000000102
Atrial fibrillation and flutter NOS	SNOMED	Clinical Finding	195082009
ECG: atrial fibrillation	SNOMED	Clinical Finding	142049000
Atrial Fibrillation	MeSH	Main Heading	D001281
Persistent atrial fibrillation	ICD10CM	4-char billing code	I48.1
Chronic atrial fibrillation	ICD10	ICD10 code	I48.2
Atrial fibrillation and flutter	ICD10	ICD10 Hierarchy	I48
Chronic atrial fibrillation	ICD10CM	4-char billing code	I48.2
Persistent atrial fibrillation	ICD10	ICD10 code	I48.1
OMOP Atrial Fibrillation 1	Cohort	Cohort	500002401
Atrial fibrillation and flutter	ICD10CM	3-char nonbill code	I48
Paroxysmal atrial fibrillation	ICD10CM	4-char billing code	I48.0
Paroxysmal atrial fibrillation	ICD10	ICD10 code	I48.0

Persistent atrial fibrillation	Read	Read	G573500
Paroxysmal atrial fibrillation	Read	Read	G573200
Atrial fibrillation and flutter NOS	Read	Read	G573z00
Chronic atrial fibrillation	Read	Read	G573700
ECG: atrial fibrillation	Read	Read	3272
Persistent Atrial Fibrillation	CIEL	Diagnosis	152500
Permanent Atrial Fibrillation	CIEL	Diagnosis	152501
Atrial Fibrillation	CIEL	Diagnosis	148203
Permanent atrial fibrillation	SNOMED	Clinical Finding	440028005
Longstanding persistent atrial fibrillation	SNOMED	Clinical Finding	706923002
Atrial fibrillation and flutter	SNOMED	Clinical Finding	195080001
Chronic atrial fibrillation	SNOMED	Clinical Finding	426749004
Transient cerebral ischemia due to atrial fibrillation	SNOMED	Clinical Finding	426814001
Rapid atrial fibrillation	SNOMED	Clinical Finding	314208002
Paroxysmal atrial fibrillation	SNOMED	Clinical Finding	282825002
Paroxysmal atrial fibrillation	SNOMED	Clinical Finding	195081002

Table S2A. Anti-arrythmic Medications

Medication Name	Generic Name	# of Patients	# of Records
AMIODARONE (BULK) MISC	Amiodarone	12	21
AMIODARONE 100 MG TABLET	Amiodarone	1,721	26,134
AMIODARONE 200 MG TABLET	Amiodarone	12,540	481,146
AMIODARONE 300 MG TABLET	Amiodarone	20	42
AMIODARONE 400 MG TABLET	Amiodarone	2,193	6,941
AMIODARONE 50 MG TAB (HALF-TAB)	Amiodarone	39	751
AMIODARONE PO	Amiodarone	310	550
CHCO FLECAINIDE 150 MG TABLET	CHCO FLECAINIDE 150 MG TABLET	23	55
DISOPYRAMIDE PHOSPHATE 100 MG CAPSULE	disopyramide phosphate 100 mg capsule	96	2,434
DISOPYRAMIDE PHOSPHATE 150 MG CAPSULE	disopyramide phosphate 150 mg capsule	63	1,033
DISOPYRAMIDE PHOSPHATE ER 100 MG CAPSULE,EXTENDED RELEASE	disopyramide phosphate ER 100 mg capsule,extended release	32	239
DISOPYRAMIDE PHOSPHATE ER 150 MG CAPSULE,EXTENDED RELEASE	disopyramide phosphate ER 150 mg capsule,extended release	48	679
DOFETILIDE 125 MCG CAPSULE	dofetilide 125 mcg capsule	488	13,712
DOFETILIDE 250 MCG CAPSULE	dofetilide 250 mcg capsule	1,075	30,176
DOFETILIDE 500 MCG CAPSULE	dofetilide 500 mcg capsule	958	18,190
DRONEDARONE 400 MG TABLET	dronedarone 400 mg tablet	1,565	21,850
FLECAINIDE 100 MG TABLET	flecainide 100 mg tablet	3,552	55,552
FLECAINIDE 150 MG TABLET	flecainide 150 mg tablet	948	5,936
FLECAINIDE 25 MG TAB (HALF-TAB)	flecainide 50 mg tablet	92	1,702
FLECAINIDE 50 MG TAB (HALF-TAB)	flecainide 100 mg tablet	24	173
FLECAINIDE 50 MG TABLET	flecainide 50 mg tablet	2,921	45,443
FLECAINIDE ORAL SOLUTION 5 MG/ML	flecainide 100 mg tablet	13	579
FLECAINIDE PO	flecainide oral	245	465
MEXILETINE 150 MG CAPSULE	mexiletine 150 mg capsule	561	37,696
MEXILETINE 200 MG CAPSULE	mexiletine 200 mg capsule	203	13,387
MEXILETINE 250 MG CAPSULE	mexiletine 250 mg capsule	78	9,734
MULTAQ 400 MG TABLET	dronedarone 400 mg tablet	890	6,646
MULTAQ PO	dronedarone oral	75	145
NORPACE 100 MG CAPSULE	disopyramide phosphate 100 mg capsule	22	288

NORPACE 150 MG CAPSULE	disopyramide phosphate 150 mg capsule	13	41
NORPACE CR 100 MG CAPSULE,EXTENDED RELEASE	disopyramide phosphate ER 100 mg capsule,extended release	16	63
NORPACE CR 150 MG CAPSULE,EXTENDED RELEASE	disopyramide phosphate ER 150 mg capsule,extended release	15	128
PACERONE 100 MG TABLET	Amiodarone	408	2,635
PACERONE 200 MG TABLET	Amiodarone	2,153	21,614
PACERONE 400 MG TABLET	Amiodarone	21	36
PACERONE PO	Amiodarone	16	31
PROPAFENONE 150 MG TABLET	propafenone 150 mg tablet	923	18,244
PROPAFENONE 225 MG TABLET	propafenone 225 mg tablet	302	1,725
PROPAFENONE 300 MG TABLET	propafenone 300 mg tablet	120	540
PROPAFENONE 75 MG TAB (HALF-TAB)	propafenone 150 mg Tab	98	3,289
PROPAFENONE ER 225 MG CAPSULE,EXTENDED RELEASE 12 HR	propafenone ER 225 mg capsule,extended release 12 hr	457	5,630
PROPAFENONE ER 325 MG CAPSULE,EXTENDED RELEASE 12 HR	propafenone ER 325 mg capsule,extended release 12 hr	190	2,707
PROPAFENONE ER 425 MG CAPSULE,EXTENDED RELEASE 12 HR	propafenone ER 425 mg capsule,extended release 12 hr	58	346
PROPAFENONE PO	propafenone oral	47	86
RYTHMOL PO	propafenone oral	25	49
RYTHMOL SR 225 MG CAPSULE,EXTENDED RELEASE	propafenone ER 225 mg capsule,extended release 12 hr	20	76
RYTHMOL SR 325 MG CAPSULE,EXTENDED RELEASE	propafenone ER 325 mg capsule,extended release 12 hr	12	44
SORINE 80 MG TABLET	sotaloL 80 mg tablet	685	4,799
SOTALOL 120 MG TABLET	sotaloL 120 mg tablet	1,190	19,983
SOTALOL 160 MG TABLET	sotaloL 160 mg tablet	252	1,334
SOTALOL 20 MG TAB (QUARTER-TAB)	sotaloL 80 mg tablet	10	206
SOTALOL 240 MG TABLET	sotaloL 240 mg tablet	21	166
SOTALOL 40 MG TAB (HALF-TAB)	sotaloL 80 mg tablet	806	26,000
SOTALOL 60 MG TAB (HALF-TAB)	sotaloL 120 mg tablet	38	429
SOTALOL 80 MG TABLET	sotaloL 80 mg tablet	3,760	72,541
SOTALOL AF 120 MG TABLET	sotaloL 120 mg tablet	38	137
SOTALOL AF 80 MG TABLET	sotaloL 80 mg tablet	107	385
SOTALOL ORAL SUSPENSION 5 MG/ML	sotaloL 80 mg tablet	38	665
SOTALOL PO	sotalol oral	193	367
TAMBOCOR PO	flecainide oral	11	21

TIKOSYN 125 MCG CAPSULE	dofetilide 125 mcg capsule	232	1,913
TIKOSYN 250 MCG CAPSULE	dofetilide 250 mcg capsule	686	5,266
TIKOSYN 500 MCG CAPSULE	dofetilide 500 mcg capsule	534	3,390
TIKOSYN PO	dofetilide oral	41	84

Table S2B. Nodal Agents

Medication Name	Generic Name	Pharmaceutical Subclass	# of Patients	# of Records
METOPROLOL TARTRATE 25 MG TABLET	metoprolol tartrate 25 mg tablet	Beta Blockers Cardiac Selective	72,126	1,517,740
METOPROLOL SUCCINATE ER 25 MG TABLET,EXTENDED RELEASE 24 HR	metoprolol succinate ER 25 mg tablet,extended release 24 hr	Beta Blockers Cardiac Selective	49,833	574,116
METOPROLOL TARTRATE 50 MG TABLET	metoprolol tartrate 50 mg tablet	Beta Blockers Cardiac Selective	41,480	1,118,028
METOPROLOL SUCCINATE ER 50 MG TABLET,EXTENDED RELEASE 24 HR	metoprolol succinate ER 50 mg tablet,extended release 24 hr	Beta Blockers Cardiac Selective	39,312	546,639
METOPROLOL TARTRATE 12.5 MG (HALF TAB)	metoprolol tartrate 25 mg tablet	Beta Blockers Cardiac Selective	21,762	708,893
ATENOLOL 25 MG TABLET	atenoloL 25 mg tablet	Beta Blockers Cardiac Selective	17,952	192,416
PROPRANOLOL 10 MG TABLET	propranoloL 10 mg tablet	Beta Blockers Non-Cardiac Selective	17,804	420,869
ATENOLOL 50 MG TABLET	atenoloL 50 mg tablet	Beta Blockers Cardiac Selective	17,024	217,101
METOPROLOL SUCCINATE ER 100 MG TABLET,EXTENDED RELEASE 24 HR	metoprolol succinate ER 100 mg tablet,extended release 24 hr	Beta Blockers Cardiac Selective	16,472	102,012
PROPRANOLOL 20 MG TABLET	propranoloL 20 mg tablet	Beta Blockers Non-Cardiac Selective	12,559	97,042
DILTIAZEM CD 120 MG CAPSULE,EXTENDED RELEASE 24 HR	dilTIAZem CD 120 mg capsule,extended release 24 hr	Calcium Channel Blockers - Benzothiazepines	10,800	154,960
DIGOXIN 125 MCG (0.125 MG) TABLET	digoxin 125 mcg (0.125 mg) tablet	Digitalis Glycosides	10,247	203,661
METOPROLOL TARTRATE 100 MG TABLET	metoprolol tartrate 100 mg tablet	Beta Blockers Cardiac Selective	10,228	53,304
TOPROL XL 50 MG TABLET,EXTENDED RELEASE	metoprolol succinate ER 50 mg tablet,extended release 24 hr	Beta Blockers Cardiac Selective	9,500	49,759
METOPROLOL TARTRATE PO	metoprolol tartrate oral	Beta Blockers Cardiac Selective	7,820	14,761
NEBIVOLOL 5 MG TABLET	nebivoloL 5 mg tablet	Beta Blockers Cardiac Selective	7,422	66,115

DILTIAZEM CD 180 MG CAPSULE,EXTENDED RELEASE 24 HR	dilTIAZem CD 180 mg capsule,extended release 24 hr	Calcium Channel Blockers - Benzothiazepines	7,103	114,582
PROPRANOLOL 40 MG TABLET	propranoloL 40 mg tablet	Beta Blockers Non-Cardiac Selective	6,367	129,419
DILTIAZEM CD 240 MG CAPSULE,EXTENDED RELEASE 24 HR	dilTIAZem CD 240 mg capsule,extended release 24 hr	Calcium Channel Blockers - Benzothiazepines	6,170	75,681
ATENOLOL 100 MG TABLET	atenoloL 100 mg tablet	Beta Blockers Cardiac Selective	6,149	34,053
TOPROL XL 25 MG TABLET,EXTENDED RELEASE	metoprolol succinate ER 25 mg tablet,extended release 24 hr	Beta Blockers Cardiac Selective	5,922	24,886
DILTIAZEM 30 MG TABLET	dilTIAZem 30 mg tablet	Calcium Channel Blockers - Benzothiazepines	5,886	175,745
METOPROLOL SUCCINATE PO	metoprolol succinate oral	Beta Blockers Cardiac Selective	5,392	10,205
METOPROLOL SUCCINATE ER 12.5 MG TAB (HALF-TAB)	metoprolol succinate ER 25 mg tablet,extended release 24 hr	Beta Blockers Cardiac Selective	4,026	77,779
PROPRANOLOL ER 60 MG CAPSULE,24 HR,EXTENDED RELEASE	propranoloL ER 60 mg capsule,24 hr,extended release	Beta Blockers Non-Cardiac Selective	3,932	31,620
NEBIVOLOL 10 MG TABLET	nebivoloL 10 mg tablet	Beta Blockers Cardiac Selective	3,878	19,011
VERAPAMIL ER (SR) 120 MG TABLET,EXTENDED RELEASE	verapamiL ER (SR) 120 mg tablet,extended release	Calcium Channel Blockers - Phenylakylamines	3,778	38,998
ATENOLOL PO	atenolol oral	Beta Blockers Cardiac Selective	3,635	6,989
VERAPAMIL ER (SR) 240 MG TABLET,EXTENDED RELEASE	verapamiL ER (SR) 240 mg tablet,extended release	Calcium Channel Blockers - Phenylakylamines	3,532	34,871
DIGOXIN 250 MCG (0.25 MG) TABLET	digoxin 250 mcg (0.25 mg) tablet	Digitalis Glycosides	3,351	29,681
DILTIAZEM 60 MG TABLET	dilTIAZem 60 mg tablet	Calcium Channel Blockers - Benzothiazepines	3,227	94,419
BYSTOLIC 5 MG TABLET	nebivoloL 5 mg tablet	Beta Blockers Cardiac Selective	3,209	17,647

PROPRANOLOL ER 80 MG CAPSULE,24 HR,EXTENDED RELEASE	propranoloL ER 80 mg capsule,24 hr,extended release	Beta Blockers Non-Cardiac Selective	2,722	26,897
BISOPROLOL FUMARATE 5 MG TABLET	bisoprolol fumarate 5 mg tablet	Beta Blockers Cardiac Selective	2,696	39,200
METOPROLOL SUCCINATE ER 200 MG TABLET,EXTENDED RELEASE 24 HR	metoprolol succinate ER 200 mg tablet,extended release 24 hr	Beta Blockers Cardiac Selective	2,596	14,947
VERAPAMIL ER (SR) 180 MG TABLET,EXTENDED RELEASE	verapamiL ER (SR) 180 mg tablet,extended release	Calcium Channel Blockers - Phenylakylamines	2,502	28,836
VERAPAMIL 40 MG TABLET	verapamiL 40 mg tablet	Antiarrhythmic - Class IV	2,241	28,059
LANOXIN 125 MCG (0.125 MG) TABLET	digoxin 125 mcg (0.125 mg) tablet	Digitalis Glycosides	2,181	14,201
VERAPAMIL 120 MG TABLET	verapamiL 120 mg tablet	Antiarrhythmic - Class IV	2,069	18,499
PROPRANOLOL PO	propranolol oral	Beta Blockers Non-Cardiac Selective	2,012	3,921
PROPRANOLOL 80 MG TABLET	propranoloL 80 mg tablet	Beta Blockers Non-Cardiac Selective	1,861	7,550
PROPRANOLOL 60 MG TABLET	propranoloL 60 mg tablet	Beta Blockers Non-Cardiac Selective	1,838	7,474
BYSTOLIC 10 MG TABLET	nebivoloL 10 mg tablet	Beta Blockers Cardiac Selective	1,699	6,678
NADOLOL 20 MG TABLET	nadoloL 20 mg tablet	Beta Blockers Non-Cardiac Selective	1,624	27,120
VERAPAMIL 80 MG TABLET	verapamiL 80 mg tablet	Antiarrhythmic - Class IV	1,570	12,134
PROPRANOLOL ER 120 MG CAPSULE,24 HR,EXTENDED RELEASE	propranoloL ER 120 mg capsule,24 hr,extended release	Beta Blockers Non-Cardiac Selective	1,506	10,632
NEBIVOLOL 20 MG TABLET	nebivoloL 20 mg tablet	Beta Blockers Cardiac Selective	1,411	6,799
NEBIVOLOL 2.5 MG TABLET	nebivoloL 2.5 mg tablet	Beta Blockers Cardiac Selective	1,381	11,659
DILTIAZEM 120 MG TABLET	dilTIAZem 120 mg tablet	Calcium Channel Blockers - Benzothiazepines	1,373	7,279
DILTIAZEM ER 60 MG CAPSULE,EXTENDED RELEASE 12 HR	dilTIAZem ER 60 mg capsule,extended release 12 hr	Calcium Channel Blockers - Benzothiazepines	1,296	23,230

DILTIAZEM ER (XR/XT) 120 MG CAPSULE,EXTENDED RELEASE 24 HR, CONTROLLED	dilTIAZem ER (XR/XT) 120 mg capsule,extended release 24 hr, controlled	Calcium Channel Blockers - Benzothiazepines	1,196	4,881
DILTIAZEM CD 360 MG CAPSULE,EXTENDED RELEASE 24 HR	dilTIAZem CD 360 mg capsule,extended release 24 hr	Calcium Channel Blockers - Benzothiazepines	1,189	5,106
DILTIAZEM CD 300 MG CAPSULE,EXTENDED RELEASE 24 HR	dilTIAZem CD 300 mg capsule,extended release 24 hr	Calcium Channel Blockers - Benzothiazepines	1,149	13,309
CARTIA XT 120 MG CAPSULE,EXTENDED RELEASE	dilTIAZem CD 120 mg capsule,extended release 24 hr	Calcium Channel Blockers - Benzothiazepines	1,133	4,002
DILTIAZEM ER (XR/XT) 180 MG CAPSULE,EXTENDED RELEASE 24 HR, CONTROLLED	dilTIAZem ER (XR/XT) 180 mg capsule,extended release 24 hr, controlled	Calcium Channel Blockers - Benzothiazepines	978	4,630
DIGOXIN 62.5 MCG TAB (HALF-TAB)	digoxin 62.5 mcg (0.0625 mg) tablet	Digitalis Glycosides	950	34,731
BISOPROLOL FUMARATE 10 MG TABLET	bisoprolol fumarate 10 mg tablet	Beta Blockers Cardiac Selective	939	5,749
DILTIAZEM ER (XR/XT) 240 MG CAPSULE,EXTENDED RELEASE 24 HR, CONTROLLED	dilTIAZem ER (XR/XT) 240 mg capsule,extended release 24 hr, controlled	Calcium Channel Blockers - Benzothiazepines	936	5,306
DILTIAZEM ER 360 MG CAPSULE,24 HR,EXTENDED RELEASE	dilTIAZem ER 360 mg capsule,24 hr,extended release	Calcium Channel Blockers - Benzothiazepines	894	4,899
NADOLOL 40 MG TABLET	nadoloL 40 mg tablet	Beta Blockers Non-Cardiac Selective	879	7,187
DILTIAZEM 90 MG TABLET	dilTIAZem 90 mg tablet	Calcium Channel Blockers - Benzothiazepines	877	19,914
DIGOX 125 MCG (0.125 MG) TABLET	digoxin 125 mcg (0.125 mg) tablet	Digitalis Glycosides	809	3,283
VERAPAMIL ER 120 MG 24 HR CAPSULE,EXTENDED RELEASE	verapamiL ER 120 mg 24 hr capsule,extended release	Calcium Channel Blockers - Phenylakylamines	795	5,829
CARTIA XT 240 MG CAPSULE,EXTENDED RELEASE	dilTIAZem CD 240 mg	Calcium Channel Blockers - Benzothiazepines	786	3,109

	capsule,extended release 24 hr			
BYSTOLIC PO	nebivolol oral	Beta Blockers Cardiac Selective	780	1,520
CARTIA XT 180 MG CAPSULE,EXTENDED RELEASE	dilTIAZem CD 180 mg capsule,extended release 24 hr	Calcium Channel Blockers - Benzothiazepines	766	2,693
VERAPAMIL ER 240 MG 24 HR CAPSULE,EXTENDED RELEASE	verapamiL ER 240 mg 24 hr capsule,extended release	Calcium Channel Blockers - Phenylakylamines	763	4,364
ATENOLOL 12.5 MG TAB (HALF-TAB)	ATENOLOL 12.5 MG TAB (HALF-TAB)	Beta Blockers Cardiac Selective	742	15,344
BYSTOLIC 2.5 MG TABLET	nebivoloL 2.5 mg tablet	Beta Blockers Cardiac Selective	726	3,556
DILTIAZEM HCL PO	diltiazem oral	Calcium Channel Blockers - Benzothiazepines	709	1,332
PROPRANOLOL ER 160 MG CAPSULE,24 HR,EXTENDED RELEASE	propranoloL ER 160 mg capsule,24 hr,extended release	Beta Blockers Non-Cardiac Selective	702	4,467
DILTIAZEM ER 120 MG CAPSULE,24 HR,EXTENDED RELEASE	dilTIAZem ER 120 mg capsule,24 hr,extended release	Calcium Channel Blockers - Benzothiazepines	684	2,451
TOPROL XL PO	metoprolol succinate oral	Beta Blockers Cardiac Selective	679	1,302
BYSTOLIC 20 MG TABLET	nebivoloL 20 mg tablet	Beta Blockers Cardiac Selective	645	2,241
DILTIAZEM ER 240 MG CAPSULE,24 HR,EXTENDED RELEASE	dilTIAZem ER 240 mg capsule,24 hr,extended release	Calcium Channel Blockers - Benzothiazepines	639	3,273
METOPROLOL TARTRATE 75 MG TABLET	metoprolol tartrate 75 mg tablet	Beta Blockers Cardiac Selective	635	1,776
DIGOXIN PO	digoxin oral	Digitalis Glycosides	579	1,065
LOPRESSOR PO	metoprolol tartrate oral	Beta Blockers Cardiac Selective	554	1,029
INDERAL LA 60 MG CAPSULE,EXTENDED RELEASE	propranoloL ER 60 mg capsule,24 hr,extended release	Beta Blockers Non-Cardiac Selective	543	2,464
TOPROL XL 100 MG TABLET,EXTENDED RELEASE	metoprolol succinate ER 100 mg tablet,extended release 24 hr	Beta Blockers Cardiac Selective	543	1,546
VERAPAMIL ER 360 MG 24 HR CAPSULE,EXTENDED RELEASE	verapamiL ER 360 mg 24 hr capsule,extended release	Calcium Channel Blockers - Phenylakylamines	526	3,389

DILTIAZEM ER 180 MG CAPSULE,24 HR,EXTENDED RELEASE	dilTIAZem ER 180 mg capsule,24 hr,extended release	Calcium Channel Blockers - Benzothiazepines	522	2,304
VERAPAMIL ER 180 MG 24 HR CAPSULE,EXTENDED RELEASE	verapamiL ER 180 mg 24 hr capsule,extended release	Calcium Channel Blockers - Phenylakylamines	441	2,291
DILTIAZEM 15 MG TAB (HALF-TAB)	dilTIAZem 30 mg tablet	Calcium Channel Blockers - Benzothiazepines	409	14,817
DILTIAZEM ORAL SUSPENSION 12 MG/ML	dilTIAZem 30 mg tablet	Calcium Channel Blockers - Benzothiazepines	385	41,619
BISOPROLOL FUMARATE 2.5 MG TAB (HALF-TAB)	bisoprolol fumarate 5 mg tablet	Beta Blockers Cardiac Selective	367	6,403
DIGOX 250 MCG (0.25 MG) TABLET	digoxin 250 mcg (0.25 mg) tablet	Digitalis Glycosides	350	1,336
DILT-XR 120 MG CAPSULE, EXTENDED RELEASE	dilTIAZem ER (XR/XT) 120 mg capsule,extended release 24 hr, controlled	Calcium Channel Blockers - Benzothiazepines	344	1,225
PROPRANOLOL 20 MG/5 ML (4 MG/ML) ORAL SOLUTION	propranoloL 20 mg/5 mL (4 mg/mL) oral solution	Beta Blockers Non-Cardiac Selective	299	23,169
PROPRANOLOL 5 MG PARTIAL TABLET	propranolol 10 mg Tab	Beta Blockers Non-Cardiac Selective	298	23,137
DIGOXIN 50 MCG/ML (0.05 MG/ML) ORAL SOLUTION	digoxin 50 mcg/mL (0.05 mg/mL) oral solution	Digitalis Glycosides	290	8,377
DIGOXIN 62.5 MCG (0.0625 MG) TABLET	digoxin 62.5 mcg (0.0625 mg) tablet	Digitalis Glycosides	284	1,075
METOPROLOL TARTRATE 37.5 MG TABLET	metoprolol tartrate 37.5 mg tablet	Beta Blockers Cardiac Selective	280	690
DILT-XR 240 MG CAPSULE, EXTENDED RELEASE	dilTIAZem ER (XR/XT) 240 mg capsule,extended release 24 hr, controlled	Calcium Channel Blockers - Benzothiazepines	266	891
DILT-XR 180 MG CAPSULE, EXTENDED RELEASE	dilTIAZem ER (XR/XT) 180 mg capsule,extended release 24 hr, controlled	Calcium Channel Blockers - Benzothiazepines	265	923
PROPRANOLOL 40 MG/5 ML (8 MG/ML) ORAL SOLUTION	propranoloL 40 mg/5 mL (8 mg/mL) oral solution	Beta Blockers Non-Cardiac Selective	261	40,490

LANOXIN 250 MCG (0.25 MG) TABLET	digoxin 250 mcg (0.25 mg) tablet	Digitalis Glycosides	245	788
DILTIAZEM ER 120 MG CAPSULE,EXTENDED RELEASE 12 HR	dilTIAZem ER 120 mg capsule,extended release 12 hr	Calcium Channel Blockers - Benzothiazepines	239	898
DILTIAZEM ER 180 MG TABLET,EXTENDED RELEASE 24 HR	dilTIAZem ER 180 mg tablet,extended release 24 hr	Calcium Channel Blockers - Benzothiazepines	236	745
VERAPAMIL ER (PM) 100 MG CAPSULE 24HR PELLET CT,EXT.RELEASE	verapamiL ER (PM) 100 mg capsule 24hr pellet CT,ext.release	Calcium Channel Blockers - Phenylakylamines	231	1,814
DILTIAZEM ER 120 MG TABLET,EXTENDED RELEASE 24 HR	dilTIAZem ER 120 mg tablet,extended release 24 hr	Calcium Channel Blockers - Benzothiazepines	223	628
ACEBUTOLOL 200 MG CAPSULE	acebutoloL 200 mg capsule	Beta Blockers Cardiac Selective, Intrinsic Sympathomimetic Activity	222	3,946
DILTIAZEM ER 240 MG TABLET,EXTENDED RELEASE 24 HR	dilTIAZem ER 240 mg tablet,extended release 24 hr	Calcium Channel Blockers - Benzothiazepines	219	861
INDERAL ORAL	propranolol oral	Beta Blockers Non-Cardiac Selective	207	401
PINDOLOL 5 MG TABLET	pindoloL 5 mg tablet	Beta Blockers Non-Cardiac Select., Intrinsic Sympathomimetic Activity	206	3,111
DILTIAZEM ER 360 MG TABLET,EXTENDED RELEASE 24 HR	dilTIAZem ER 360 mg tablet,extended release 24 hr	Calcium Channel Blockers - Benzothiazepines	177	660
DILTIAZEM 45 MG TAB (HALF-TAB)	dilTIAZem 90 mg tablet	Calcium Channel Blockers - Benzothiazepines	171	7,360
CARTIA XT 300 MG CAPSULE,EXTENDED RELEASE	dilTIAZem CD 300 mg capsule,extended release 24 hr	Calcium Channel Blockers - Benzothiazepines	162	533
VERAPAMIL ER (PM) 200 MG CAPSULE 24HR PELLET CT,EXT.RELEASE	verapamiL ER (PM) 200 mg capsule 24hr pellet CT,ext.release	Calcium Channel Blockers - Phenylakylamines	159	1,002
DILTIAZEM ER 300 MG CAPSULE,24 HR,EXTENDED RELEASE	dilTIAZem ER 300 mg capsule,24 hr,extended release	Calcium Channel Blockers - Benzothiazepines	158	862

NADOLOL 80 MG TABLET	nadoloL 80 mg tablet	Beta Blockers Non-Cardiac Selective	155	1,056
DIGITEK 125 MCG (0.125 MG) TABLET	digoxin 125 mcg (0.125 mg) tablet	Digitalis Glycosides	145	396
DILTIAZEM ER 90 MG CAPSULE,EXTENDED RELEASE 12 HR	dilTIAZem ER 90 mg capsule,extended release 12 hr	Calcium Channel Blockers - Benzothiazepines	131	1,629
BISOPROLOL FUMARATE PO	bisoprolol fumarate oral	Beta Blockers Cardiac Selective	127	245
CARTIA XT PO	diltiazem oral	Calcium Channel Blockers - Benzothiazepines	117	228
TAZTIA XT 360 MG CAPSULE,EXTENDED RELEASE	dilTIAZem ER 360 mg capsule,24 hr,extended release	Calcium Channel Blockers - Benzothiazepines	117	344
VERAPAMIL ER (PM) 300 MG CAPSULE 24HR PELLET CT,EXT.RELEASE	verapamiL ER (PM) 300 mg capsule 24hr pellet CT,ext.release	Calcium Channel Blockers - Phenylakylamines	115	729
CARDIZEM PO	diltiazem oral	Calcium Channel Blockers - Benzothiazepines	112	210
PROPRANOLOL XL 80 MG CAPSULE,EXTENDED RELEASE 24 HR	propranoloL XL 80 mg capsule,extended release 24 hr	Beta Blockers Non-Cardiac Selective	111	326
NADOLOL PO	nadolol oral	Beta Blockers Non-Cardiac Selective	110	212
LOPRESSOR 50 MG TABLET	metoprolol tartrate 50 mg tablet	Beta Blockers Cardiac Selective	109	235
PROPRANOLOL XL 120 MG CAPSULE,EXTENDED RELEASE 24 HR	propranoloL XL 120 mg capsule,extended release 24 hr	Beta Blockers Non-Cardiac Selective	105	323
METOPROLOL SUCCINATE ER 25 MG CAPSULE SPRINKLE, EXT. RELEASE 24 HR	metoprolol succinate ER 25 mg capsule sprinkle, ext. release 24 hr	Beta Blockers Cardiac Selective	101	224
INDERAL LA PO	propranolol oral	Beta Blockers Non-Cardiac Selective	100	196
METOPROLOL SUCCINATE ER 100 MG CAPSULE SPRINKLE, EXT. RELEASE 24 HR	metoprolol succinate ER 100 mg capsule sprinkle, ext. release 24 hr	Beta Blockers Cardiac Selective	84	178

NADOLOL 10 MG TAB (HALF-TAB)	nadoloL 20 mg tablet	Beta Blockers Non-Cardiac Selective	84	1,702
BISOPROLOL FUMARATE 1.25 MG TAB (QUARTER-TAB)	bisoprolol fumarate 5 mg tablet	Beta Blockers Cardiac Selective	80	1,184
METOPROLOL SUCCINATE ER 50 MG CAPSULE SPRINKLE, EXT. RELEASE 24 HR	metoprolol succinate ER 50 mg capsule sprinkle, ext. release 24 hr	Beta Blockers Cardiac Selective	72	156
CARDIZEM CD 240 MG CAPSULE,EXTENDED RELEASE	dilTIAZem CD 240 mg capsule,extended release 24 hr	Calcium Channel Blockers - Benzothiazepines	71	161
VERAPAMIL 60 MG TAB (HALF-TAB)	verapamil 60 mg Tab	Antiarrhythmic - Class IV	70	1,375
ACEBUTOLOL 400 MG CAPSULE	acebutoloL 400 mg capsule	Beta Blockers Cardiac Selective, Intrinsic Sympathomimeti c Activity	68	623
DIGITEK 250 MCG (0.25 MG) TABLET	digoxin 250 mcg (0.25 mg) tablet	Digitalis Glycosides	66	201
TOPROL XL 200 MG TABLET,EXTENDED RELEASE	metoprolol succinate ER 200 mg tablet,extended release 24 hr	Beta Blockers Cardiac Selective	66	278
CARDIZEM CD PO	diltiazem oral	Calcium Channel Blockers - Benzothiazepines	64	118
TENORMIN PO	atenolol oral	Beta Blockers Cardiac Selective	61	119
DIGOX PO	digoxin oral	Digitalis Glycosides	56	107
DILTIAZEM ER 420 MG CAPSULE,24 HR,EXTENDED RELEASE	dilTIAZem ER 420 mg capsule,24 hr,extended release	Calcium Channel Blockers - Benzothiazepines	56	351
LANOXIN PO	digoxin oral	Digitalis Glycosides	54	105
VERAPAMIL 20 MG TAB (HALF-TAB)	verapamil 40 mg Tab	Antiarrhythmic - Class IV	53	833
DILTIAZEM ER 300 MG TABLET,EXTENDED RELEASE 24 HR	dilTIAZem ER 300 mg tablet,extended release 24 hr	Calcium Channel Blockers - Benzothiazepines	51	194
CALAN SR 120 MG TABLET,EXTENDED RELEASE	verapamiL ER (SR) 120 mg tablet,extended release	Calcium Channel Blockers - Phenylakylamines	50	186

CARDIZEM CD 120 MG CAPSULE,EXTENDED RELEASE	dilTIAZem CD 120 mg capsule,extended release 24 hr	Calcium Channel Blockers - Benzothiazepines	50	97
METOPROLOL 10 MG/ML ORAL SUSPENSION (COMPOUNDING KIT)	Metoprolol 10 MG/ML Oral Suspension	Beta Blockers Cardiac Selective	49	2,093
DIGOXIN 0.25 MG/5 ML (250 MCG/5 ML) (50 MCG/ML) (5 ML) ORAL SOLUTION	digoxin 0.25 mg/5 mL (250 mcg/5 mL) (50 mcg/mL) (5 mL) oral solution	Digitalis Glycosides	48	98
TAZTIA XT 180 MG CAPSULE,EXTENDED RELEASE	dilTIAZem ER 180 mg capsule,24 hr,extended release	Calcium Channel Blockers - Benzothiazepines	48	194
TAZTIA XT 120 MG CAPSULE,EXTENDED RELEASE	dilTIAZem ER 120 mg capsule,24 hr,extended release	Calcium Channel Blockers - Benzothiazepines	45	139
METOPROLOL TARTRATE 6.25 MG (QUARTER TAB)	metoprolol tartrate 25 mg tablet	Beta Blockers Cardiac Selective	44	1,162
TIAZAC 120 MG CAPSULE,EXTENDED RELEASE	dilTIAZem ER 120 mg capsule,24 hr,extended release	Calcium Channel Blockers - Benzothiazepines	44	114
CALAN SR 240 MG TABLET,EXTENDED RELEASE	verapamiL ER (SR) 240 mg tablet,extended release	Calcium Channel Blockers - Phenylakylamines	43	120
FIRST-METOPROLOL 10 COMPOUNDING KIT	Metoprolol 10 MG/ML Oral Suspension	Beta Blockers Cardiac Selective	43	697
CARDIZEM CD 180 MG CAPSULE,EXTENDED RELEASE	dilTIAZem CD 180 mg capsule,extended release 24 hr	Calcium Channel Blockers - Benzothiazepines	42	91
CARDIZEM CD 360 MG CAPSULE,EXTENDED RELEASE	dilTIAZem CD 360 mg capsule,extended release 24 hr	Calcium Channel Blockers - Benzothiazepines	41	87
DILTIAZEM ER 420 MG TABLET,EXTENDED RELEASE 24 HR	dilTIAZem ER 420 mg tablet,extended release 24 hr	Calcium Channel Blockers - Benzothiazepines	41	146
LOPRESSOR 100 MG TABLET	metoprolol tartrate 100 mg tablet	Beta Blockers Cardiac Selective	41	95
TENORMIN 50 MG TABLET	atenoloL 50 mg tablet	Beta Blockers Cardiac Selective	40	138
PINDOLOL 2.5 MG TAB (HALF-TAB)	pindolol 2.5 mg Tab	Beta Blockers Non-Cardiac Select., Intrinsic Sympathomimetic Activity	39	465

TAZTIA XT 240 MG CAPSULE,EXTENDED RELEASE	dilTIAZem ER 240 mg capsule,24 hr,extended release	Calcium Channel Blockers - Benzothiazepines	39	135
DIGOXIN 100 MCG (0.1 MG) CAPSULE	digoxin 100 mcg (0.1 mg) capsule	Digitalis Glycosides	38	76
PINDOLOL 10 MG TABLET	pindoloL 10 mg tablet	Beta Blockers Non-Cardiac Select., Intrinsic Sympathomimetic Activity	38	241
VERAPAMIL 40 MG (HALF-TAB)	verapamil 40 mg partial tablet	Antiarrhythmic - Class IV	34	981
CARDIZEM LA 120 MG TABLET,EXTENDED RELEASE	dilTIAZem ER 120 mg tablet,extended release 24 hr	Calcium Channel Blockers - Benzothiazepines	33	99
TENORMIN 25 MG TABLET	atenoloL 25 mg tablet	Beta Blockers Cardiac Selective	33	94
TIMOLOL MALEATE PO	timolol oral	Beta Blockers Non-Cardiac Selective	32	63
DIGITEK PO	digoxin oral	Digitalis Glycosides	31	61
TIAZAC PO	diltiazem oral	Calcium Channel Blockers - Benzothiazepines	31	57
DILTIA XT PO	diltiazem oral	Calcium Channel Blockers - Benzothiazepines	30	52
MHS PEDS ATENOLOL 10 MG/ML ORAL SUSP	atenoloL 100 mg tablet	Beta Blockers Cardiac Selective	29	136
TIAZAC 240 MG CAPSULE,EXTENDED RELEASE	dilTIAZem ER 240 mg capsule,24 hr,extended release	Calcium Channel Blockers - Benzothiazepines	28	66
TIMOLOL 10 MG TABLET	timoloL 10 mg tablet	Beta Blockers Non-Cardiac Selective	27	159
VERAPAMIL SR 90 MG TAB (HALF-TAB)	verapamil SR 180 mg Tab	Calcium Channel Blockers - Phenylakylamines	27	234
DILT-CD 120 MG CAPSULE,EXTENDED RELEASE	dilTIAZem CD 120 mg capsule,extended release 24 hr	Calcium Channel Blockers - Benzothiazepines	26	52
INDERAL LA 80 MG CAPSULE,EXTENDED RELEASE	propranoloL ER 80 mg capsule,24 hr,extended release	Beta Blockers Non-Cardiac Selective	26	88
MATZIM LA 240 MG TABLET,EXTENDED RELEASE	dilTIAZem ER 240 mg tablet,extended release 24 hr	Calcium Channel Blockers - Benzothiazepines	25	91

BETAXOLOL 10 MG TABLET	betaxoloL 10 mg tablet	Beta Blockers Cardiac Selective	23	83
NEBIVOLOL PO	nebivolol oral	Beta Blockers Cardiac Selective	23	43
TIAZAC 180 MG CAPSULE,EXTENDED RELEASE	dilTIAZem ER 180 mg capsule,24 hr,extended release	Calcium Channel Blockers - Benzothiazepines	22	46
DIGOXIN 50 MCG (0.05 MG) CAPSULE	digoxin 50 mcg (0.05 mg) capsule	Digitalis Glycosides	21	47
MATZIM LA 180 MG TABLET,EXTENDED RELEASE	dilTIAZem ER 180 mg tablet,extended release 24 hr	Calcium Channel Blockers - Benzothiazepines	21	74
CARDIZEM 120 MG TABLET	dilTIAZem 120 mg tablet	Calcium Channel Blockers - Benzothiazepines	20	40
CARDIZEM LA 180 MG TABLET,EXTENDED RELEASE	dilTIAZem ER 180 mg tablet,extended release 24 hr	Calcium Channel Blockers - Benzothiazepines	20	43
DILT-CD 180 MG CAPSULE,EXTENDED RELEASE	dilTIAZem CD 180 mg capsule,extended release 24 hr	Calcium Channel Blockers - Benzothiazepines	20	40
INDERAL LA 120 MG CAPSULE,EXTENDED RELEASE	propranoloL ER 120 mg capsule,24 hr,extended release	Beta Blockers Non-Cardiac Selective	20	62
TIAZAC 360 MG CAPSULE,EXTENDED RELEASE	dilTIAZem ER 360 mg capsule,24 hr,extended release	Calcium Channel Blockers - Benzothiazepines	20	46
VERAPAMIL SR 60 MG TAB (HALF-TAB)	verapamil SR 120 mg Tab	Calcium Channel Blockers - Phenylakylamines	20	163
NADOLOL 120 MG TABLET	nadoloL 120 mg tablet	Beta Blockers Non-Cardiac Selective	19	45
CARDIZEM LA 240 MG TABLET,EXTENDED RELEASE	dilTIAZem ER 240 mg tablet,extended release 24 hr	Calcium Channel Blockers - Benzothiazepines	17	41
DILT-XR PO	diltiazem oral	Calcium Channel Blockers - Benzothiazepines	17	32
INDERAL XL PO	propranolol oral	Beta Blockers Non-Cardiac Selective	17	33
METOPROLOL SUCCINATE ER 200 MG CAPSULE SPRINKLE, EXT. RELEASE 24 HR	metoprolol succinate ER 200 mg capsule sprinkle, ext. release 24 hr	Beta Blockers Cardiac Selective	17	43

VERAPAMIL 10 MG TAB (PARTIAL TAB)	verapamil 40 mg tablet	Antiarrhythmic - Class IV	17	66
DIGOXIN 187.5 MCG (0.1875 MG) TABLET	digoxin 187.5 mcg (0.1875 mg) tablet	Digitalis Glycosides	16	44
TAZTIA XT 300 MG CAPSULE,EXTENDED RELEASE	dilTIAZem ER 300 mg capsule,24 hr,extended release	Calcium Channel Blockers - Benzothiazepines	16	55
CORGARD PO	nadolol oral	Beta Blockers Non-Cardiac Selective	15	28
TIMOLOL 5 MG TABLET	timoloL 5 mg tablet	Beta Blockers Non-Cardiac Selective	15	269
DILTIAZEM MALATE PO	diltiazem malate oral	Calcium Channel Blockers - Benzothiazepines	14	26
MATZIM LA 360 MG TABLET,EXTENDED RELEASE	dilTIAZem ER 360 mg tablet,extended release 24 hr	Calcium Channel Blockers - Benzothiazepines	14	46
TAZTIA XT PO	diltiazem oral	Calcium Channel Blockers - Benzothiazepines	14	26
TENORMIN 100 MG TABLET	atenoloL 100 mg tablet	Beta Blockers Cardiac Selective	14	31
ACEBUTOLOL PO	acebutolol oral	Beta Blockers Cardiac Selective, Intrinsic Sympathomimetic Activity	13	23
INDERAL LA 160 MG CAPSULE,EXTENDED RELEASE	propranoloL ER 160 mg capsule,24 hr,extended release	Beta Blockers Non-Cardiac Selective	13	41
VERAPAMIL ORAL SUSPENSION 50 MG/ML	verapamiL 80 mg tablet	Antiarrhythmic - Class IV	12	696
ZEBETA PO	bisoprolol fumarate oral	Beta Blockers Cardiac Selective	12	23
DIGOXIN 200 MCG (0.2 MG) CAPSULE	digoxin 200 mcg (0.2 mg) capsule	Digitalis Glycosides	11	23
DIGOXIN 500 MCG (0.5 MG) TABLET	digoxin 500 mcg (0.5 mg) tablet	Digitalis Glycosides	11	22
MATZIM LA PO	diltiazem oral	Calcium Channel Blockers - Benzothiazepines	11	22
BETAXOLOL 20 MG TABLET	betaxoloL 20 mg tablet	Beta Blockers Cardiac Selective	10	31
DILT-CD 240 MG CAPSULE,EXTENDED RELEASE	dilTIAZem CD 240 mg capsule,extended release 24 hr	Calcium Channel Blockers - Benzothiazepines	10	21

DILT-CD 300 MG CAPSULE,EXTENDED RELEASE	dilTIAZem CD 300 mg capsule,extended release 24 hr	Calcium Channel Blockers - Benzothiazepines	10	20

Table S2C. Ablation Codes

Procedure Patient Friendly Name	(Billing) Procedure Key	Other Code Type	# Unique Pt.
CEP ATRIAL FIBRILLATION ABLATION	69792	CV1013	1,419
CEP ATRIAL FLUTTER ABLATION	69786	CV1007	810
CEP ATRIAL FLUTTER ABLATION	108581	*Not Applicable	984
CEP ATRIAL FLUTTER ABLATION W/TEE	107857	*Not Applicable	281

Table S2D. Cardioversion Codes

Procedure Name	(Billing) Procedure Key	Cpt Code	# Unique Pt.
ATRIAL CARDIOVERSION	250377		372
CARDIOVERSION	122746	92960	13
CARDIOVERSION	62311	92960	32
CARDIOVERSION	62346	92960	161
CARDIOVERSION	29341	92960	605
CARDIOVERSION	128525	92960	902
CARDIOVERSION	128490	92960	1,037
CARDIOVERSION	54063		2,980
CARDIOVERSION	107092	92960	3,062
CARDIOVERSION ELECTIVE ARRHYTHMIA EXTERNAL	9812	92960	252
CARDIOVERSION ELECTIVE ARRHYTHMIA EXTERNAL	96050	92960	3,606
CARDIOVERSION ELECTIVE ARRHYTHMIA EXTERNAL	247096	92960	4,247
CARDIOVERSION ELECTIVE ARRHYTHMIA INTERNAL SPX	254162	92961	157
CARDIOVERSION, ELECTIVE;EXTERN	43401		171
CEP CARDIOVERSION	1818		1,706
CEP CARDIOVERSION	107670	*Not Applicable	2,443
CEP CARDIOVERSION W/TEE PRIOR	108463	*Not Applicable	2,322
ED CARDIOVERSION PROCEDURE	68238	ED2020	1,648
EP-CARDIOVERSION	63929	92960	76
EP-CARDIOVERSION	129315	92960	1,286
Ibutilide Enhanced Cardioversion	70803		17
PR ANES CARDIOVERSION, ELECTIVE;EXTERN	173895	A92960	21

TRANSESOPHAGEAL WITH CARDIOVERSION	64617	2,547

Figure S1. Learning curve for combined SMOTE model

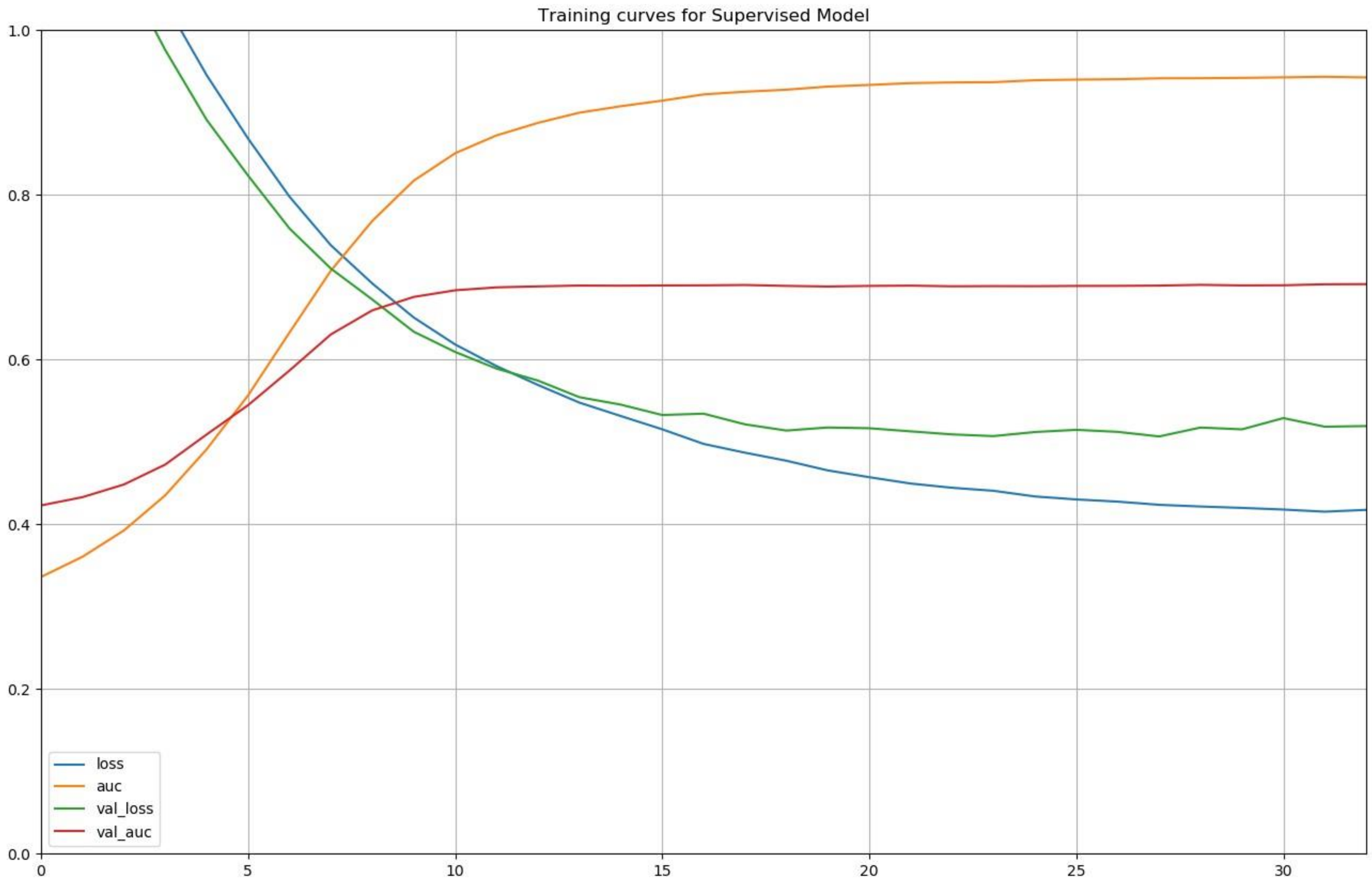

Table S3A

RANDOM FOREST	F1 score	AUC	Accuracy	Recall	Precision	Optimal Hyperparameters
None	0.005	0.500	0.923	0.003	0.077	Estimators: 200<br>Max features: 8<br>Max leaf nodes: 300
Random Undersampling	0.183	0.604	0.578	0.634	0.107	Estimators: 500<br>Max features: 3<br>Max leaf nodes: 50
Random Oversampling	0.179	0.591	0.611	0.568	0.106	Estimators: 1000<br>Max features: 6<br>Max leaf nodes: 300
SMOTE	0.186	0.591	0.689	0.476	0.116	Estimators: 200<br>Max features: 8<br>Max leaf nodes: 300
Tomek Links	0.005	0.500	0.923	0.003	0.064	Estimators: 1000<br>Max features: 8<br>Max leaf nodes: 300

Grid Search Parameters: Estimators (200, 500, 1000), Max features (3, 6, 8), Max leaf nodes (50, 100, 200, 300)

Table S3B

GRADIENT BOOSTING	F1 score	AUC	Accuracy	Recall	Precision	Optimal Hyperparameters
None	0.011	0.500	0.920	0.006	0.077	Booster: gbtree<br>Eta: 0.9<br>Gamma: 0<br>Alpha: 0<br>Lambda: 0
Random Undersampling	0.169	0.591	0.404	0.810	0.094	Booster: gbtree<br>Eta: 0.7<br>Gamma: 100<br>Alpha: 0<br>Lambda: 0
Random Oversampling	0.179	0.591	0.614	0.563	0.106	Booster: gbtree<br>Eta: 0.9<br>Gamma: 0<br>Alpha: 1<br>Lambda: 0
SMOTE	0.178	0.573	0.741	0.375	0.117	Booster: gbtree<br>Eta: 0.9<br>Gamma: 0<br>Alpha: 1<br>Lambda: 0
Tomek Links	0.010	0.500	0.922	0.005	0.085	Booster: gbtree<br>Eta: 0.7<br>Gamma: 0<br>Alpha: 0<br>Lambda: 0

Grid Search Parameters: Boosters (gbtree, gblinear, dart); Eta (0.1, 0.3, 0.7, 0.9); Gamma (0, 5, 10, 100); Alpha (0, 1); Lambda (0,1)

Table S3C

KNN	F1 score	AUC	Accuracy	Recall	Precision	Optimal Hyperparameters
None	0.105	0.518	0.877	0.097	0.115	Neighbors = 1
Random Undersampling	0.181	0.605	0.541	0.682	0.105	Neighbors = 500
Random Oversampling	0.153	0.546	0.642	0.434	0.093	Neighbors = 5
SMOTE	0.163	0.556	0.739	0.339	0.107	Neighbors = 1
Tomek Links	0.098	0.514	0.870	0.094	0.102	Neighbors = 1

Grid Search Parameters: Neighbors (1, 5, 10, 15, 20, 25, 50, 100, 500)

Table S3D

Naïve Bayes	F1 score	AUC	Accuracy	Recall	Precision	Optimal Hyperparameters
None	0.0	0.0	0.925	0.0	0.0	Alpha = 0
Random Undersampling	0.181	0.599	0.587	0.613	0.106	Alpha = 0
Random Oversampling	0.183	0.600	0.593	0.609	0.107	Alpha = 0
SMOTE	0.184	0.602	0.596	0.609	0.108	Alpha = 0
Tomek Links	0.0	0.0	0.925	0.0	0.0	Alpha = 0

Grid Search Parameters: Alpha (0, 0.1, 0.4, 0.6, 0.9, 1.0)

Table S4A. Neural Network Training Results

	Resampling	Layers	Neurons/layer	Accuracy	F1 score	Recall	Precision	AUC
NN 1	SMOTE	2	1000	0.848	0.252	0.343	0.200	0.616
NN 2	SMOTE	3	1000	0.815	0.227	0.363	0.165	0.607
NN 3	SMOTE	4	1000	0.814	0.239	0.391	0.172	0.620
NN 4	None	1	1000	0.924	0.068	0.037	0.414	0.516
NN 5	None	1	5000	0.924	0.053	0.028	0.431	0.513
NN 6	None	2	1000	0.925	0.0	0.0	0.0	0.5
NN 7	SMOTE	1	1000	0.785	0.235	0.442	0.160	0.628
NN 8	SMOTE	1	5000	0.782	0.232	0.441	0.157	0.625
NN 9	SMOTE	2	1000	0.835	0.260	0.387	0.195	0.629
NN10	Random US	1	1000	0.716	0.213	0.516	0.134	0.624
NN 11	Random US	1	5000	0.680	0.213	0.580	0.130	0.634
NN 12	Random US	2	1000	0.681	0.206	0.553	0.127	0.622

Neural Network (NN) parameters:
Elu activation (except final layer—Sigmoid); He initialization; L2 regularization (Penalty = 0.01); Dropout (20%); Batch normalization; Binary cross-entropy loss; RMS prop optimizer with learning rate 1e-4, rho = 0.9; metrics = AUC; 50 epochs with early stopping
Resampling methods: synthetic minority oversampling (SMOTE), random undersampling (US)

Table S4B. 2x2 Contingency tables for each model

NN1

	Predicted Rate	Predicted Rhythm
Actual Rate	8641	1079
Actual Rhythm	516	269

NN2

	Predicted Rate	Predicted Rhythm
Actual Rate	8279	1441
Actual Rhythm	500	285

NN3

	Predicted Rate	Predicted Rhythm
Actual Rate	8244	1476
Actual Rhythm	478	307

NN4

	Predicted Rate	Predicted Rhythm
Actual Rate	9679	41
Actual Rhythm	756	29

NN5

	Predicted Rate	Predicted Rhythm
Actual Rate	9691	29
Actual Rhythm	763	22

NN6

	Predicted Rate	Predicted Rhythm
Actual Rate	9720	0
Actual Rhythm	785	0

NN7

	Predicted Rate	Predicted Rhythm
Actual Rate	7903	1817
Actual Rhythm	438	347

NN8

	Predicted Rate	Predicted Rhythm
Actual Rate	7867	1853
Actual Rhythm	439	346

NN9

	Predicted Rate	Predicted Rhythm
Actual Rate	8469	1251
Actual Rhythm	481	304

NN10

	Predicted Rate	Predicted Rhythm
Actual Rate	7113	2607
Actual Rhythm	380	405

NN11

	Predicted Rate	Predicted Rhythm
Actual Rate	6687	3033
Actual Rhythm	330	455

NN12

	Predicted Rate	Predicted Rhythm
Actual Rate	6725	2995
Actual Rhythm	351	434

Figures S2A-D

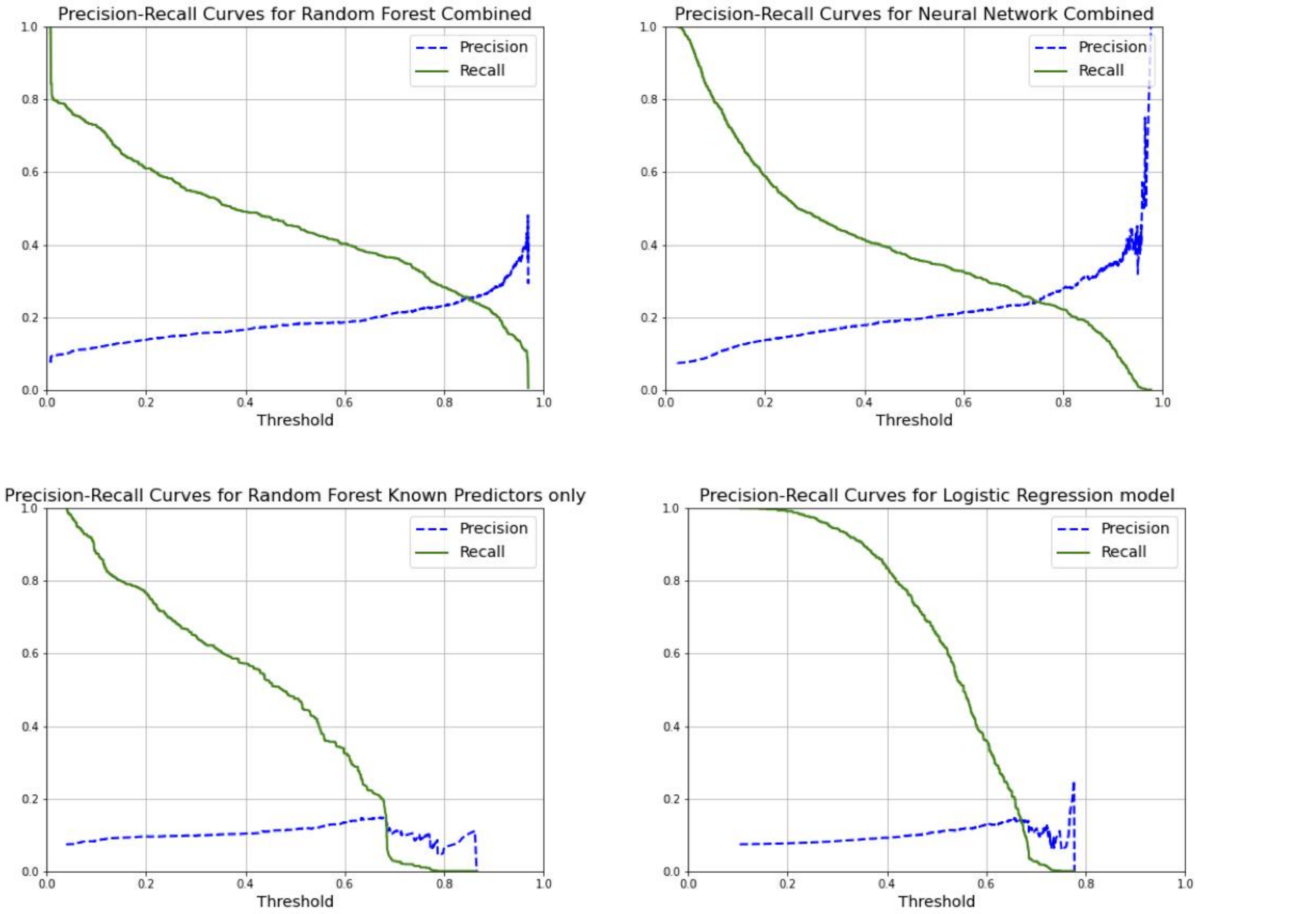

Figure S2E

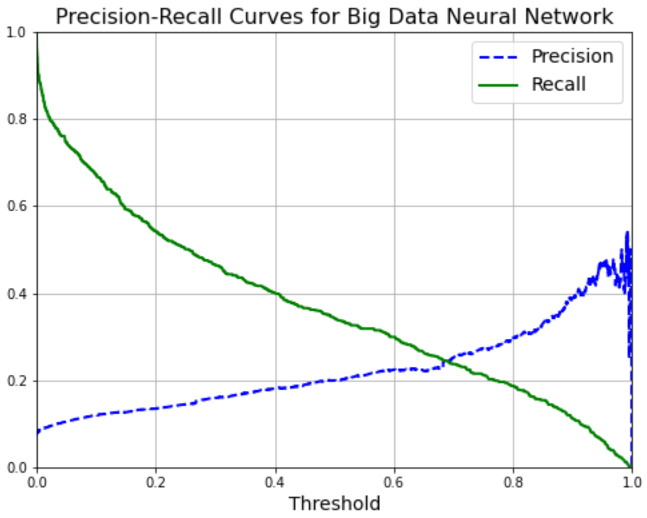
Precision-Recall Curves for Big Data Neural Network
Precision
Recall
Threshold
0.0
0.2
0.4
0.6
0.8
1.0

Table S5A. Odds ratios and statistics for regression model with Big Data Prediction

**Features**	**Chi-square (Likelihood ratio test)**	**P value**
Big Data prediction	304.42	< 0.0001
Age	77.97	< 0.0001
Obesity diagnosis	21.04	< 0.0001
Diabetes diagnosis	7.93	0.005
Sex	6.32	0.012
Hypertension diagnosis	3.26	0.071
Mitral valve disease	1.72	0.190
Heart failure diagnosis	1.43	0.231
CAD diagnosis	1.18	0.278

Table S5B. Feature Importance of Random forest model with Big Data Prediction

**Features**	**Importance**
Big Data prediction	0.8513
Age	0.1041
Mitral valve disease	0.0115
Heart failure diagnosis	0.0114
Hypertension diagnosis	0.0059
CAD diagnosis	0.0050
Sex	0.0047
Diabetes diagnosis	0.0040
Obesity diagnosis	0.0021

Table S5C. Confusion Matrices for each combined model

RF Combined Confusion Matrix

	Predicted Rate	Predicted Rhythm
Actual Rate	8119	1601
Actual Rhythm	431	354

NN Combined Confusion Matrix

	Predicted Rate	Predicted Rhythm
Actual Rate	8580	1140
Actual Rhythm	510	275

Logistic Regression with SMOTE

	Predicted Rate	Predicted Rhythm
Actual Rate	5477	4243
Actual Rhythm	272	513

Tables S6A-6E. Values for proportions of graphs

Table S6A. Neural Network Combined

Threshold	0.1	0.2	0.3	0.4	0.5	0.6	0.7	0.8	0.9
Appropriate Referrals	0.007	0.015	0.022	0.03	0.037	0.045	0.052	0.06	0.067
Inappropriate Referrals	0.014	0.037	0.079	0.131	0.213	0.287	0.389	0.552	0.741
Missed Appropriate Referrals	0.067	0.06	0.052	0.045	0.037	0.03	0.022	0.015	0.008

Table S6B. Random Forest Combined

Threshold	0.1	0.2	0.3	0.4	0.5	0.6	0.7	0.8	0.9
Appropriate Referrals	0.007	0.015	0.022	0.03	0.037	0.045	0.052	0.06	0.066
Inappropriate Referrals	0.01	0.038	0.076	0.126	0.19	0.274	0.375	0.57	0.687
Missed Appropriate Referrals	0.067	0.06	0.052	0.045	0.037	0.03	0.022	0.015	0.009

Table S6C. Random Forest Known Predictors only

Threshold	0.1	0.2	0.3	0.4	0.5	0.6	0.7	0.8	0.9
Appropriate Referrals	0.007	0.015	0.022	0.03	0.037	0.045	0.052	0.06	0.067
Inappropriate Referrals	0.046	0.087	0.136	0.214	0.296	0.39	0.483	0.58	0.735
Missed Appropriate Referrals	0.068	0.06	0.053	0.045	0.037	0.03	0.022	0.015	0.008

Table S6D. Logistic Regression Known Predictors only

Threshold	0.1	0.2	0.3	0.4	0.5	0.6	0.7	0.8	0.9
Appropriate Referrals	0.007	0.015	0.022	0.029	0.037	0.045	0.052	0.06	0.067
Inappropriate Referrals	0.046	0.088	0.151	0.212	0.281	0.352	0.45	0.571	0.688
Missed Appropriate Referrals	0.067	0.06	0.052	0.045	0.037	0.03	0.022	0.015	0.009

Table S6E. Neural Network BD Predictors only

Threshold	0.1	0.2	0.3	0.4	0.5	0.6	0.7	0.8	0.9
Appropriate Referrals	0.007	0.015	0.022	0.03	0.037	0.045	0.052	0.06	0.067
Inappropriate Referrals	0.011	0.04	0.079	0.135	0.218	0.31	0.408	0.574	0.738
Missed Appropriate Referrals	0.067	0.06	0.052	0.045	0.037	0.03	0.022	0.015	0.008

Figure S3. Select models stratified by age at time of visit.

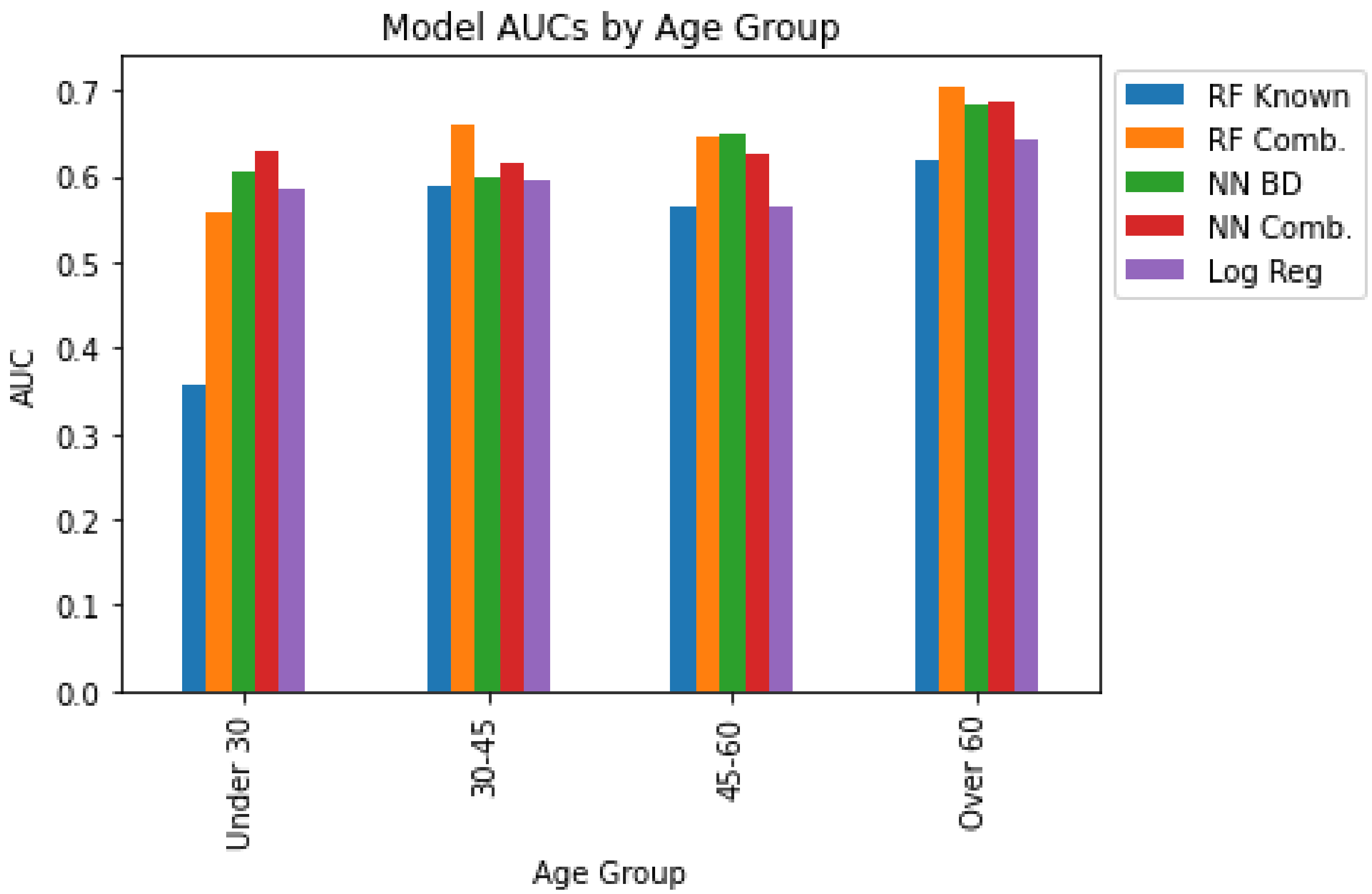

The RF Known (blue) and logistic regression (purple) models used only known predictors. The NN BD model (green) used only BD predictors. The RF Comb. (orange) and NN Comb. (red) models use both known and BD predictors. The differences in AUC between the age groups is not statistically significant (p = 0.06).

Table S7. All model, data, and hyperparameter permutations tested

#	Method	Data Input	Resampling	Hyperparameters*
1	Random Forest	Known predictors only	None	No. of estimators = [200, 500, 1000]<br>Max features = [3, 6, 8]<br>Max leaf nodes = [50, 100, 200, 300]
2	Random Forest	Known predictors only	Random US	No. of estimators = [200, 500, 1000]<br>Max features = [3, 6, 8]<br>Max leaf nodes = [50, 100, 200, 300]
3	Random Forest	Known predictors only	Random OS	No. of estimators = [200, 500, 1000]<br>Max features = [3, 6, 8]<br>Max leaf nodes = [50, 100, 200, 300]
4	Random Forest	Known predictors only	SMOTE	No. of estimators = [200, 500, 1000]<br>Max features = [3, 6, 8]<br>Max leaf nodes = [50, 100, 200, 300]
5	Random Forest	Known predictors only	Tomek Links	No. of estimators = [200, 500, 1000]<br>Max features = [3, 6, 8]<br>Max leaf nodes = [50, 100, 200, 300]
6	Random Forest	Known and BD predictors	SMOTE	No. of estimators = [200, 500, 1000]<br>Max features = [3, 6, 8]<br>Max leaf nodes = [50, 100, 200, 300]
7	Gradient Boosting	Known predictors only	None	Boosters = [gbtree, gblinear, dart]<br>Eta = [0.1, 0.3, 0.7, 0.9]<br>Gamma = [0, 5, 10, 100]<br>Alpha = [0, 1]<br>Lambda = [0,1]
8	Gradient Boosting	Known predictors only	Random US	Boosters = [gbtree, gblinear, dart]<br>Eta = [0.1, 0.3, 0.7, 0.9]<br>Gamma = [0, 5, 10, 100]<br>Alpha = [0, 1]<br>Lambda = [0,1]
9	Gradient Boosting	Known predictors only	Random OS	Boosters = [gbtree, gblinear, dart]<br>Eta = [0.1, 0.3, 0.7, 0.9]<br>Gamma = [0, 5, 10, 100]<br>Alpha = [0, 1]<br>Lambda = [0,1]
10	Gradient Boosting	Known predictors only	SMOTE	Boosters = [gbtree, gblinear, dart]<br>Eta = [0.1, 0.3, 0.7, 0.9]<br>Gamma = [0, 5, 10, 100]<br>Alpha = [0, 1]<br>Lambda = [0,1]

11	Gradient Boosting	Known predictors only	Tomek Links	Boosters = [gbtree, gblinear, dart]<br>Eta = [0.1, 0.3, 0.7, 0.9]<br>Gamma = [0, 5, 10, 100]<br>Alpha = [0, 1]<br>Lambda = [0,1]
12	K Nearest Neighbors	Known predictors only	None	Neighbors = [1, 5, 10, 15, 20, 25, 50, 100, 500]
13	K Nearest Neighbors	Known predictors only	Random US	Neighbors = [1, 5, 10, 15, 20, 25, 50, 100, 500]
14	K Nearest Neighbors	Known predictors only	Random OS	Neighbors = [1, 5, 10, 15, 20, 25, 50, 100, 500]
15	K Nearest Neighbors	Known predictors only	SMOTE	Neighbors = [1, 5, 10, 15, 20, 25, 50, 100, 500]
16	K Nearest Neighbors	Known predictors only	Tomek Links	Neighbors = [1, 5, 10, 15, 20, 25, 50, 100, 500]
17	Naïve Bayes	Known predictors only	None	Alpha = [0, 0.1, 0.4, 0.6, 0.9, 1.0]
18	Naïve Bayes	Known predictors only	Random US	Alpha = [0, 0.1, 0.4, 0.6, 0.9, 1.0]
19	Naïve Bayes	Known predictors only	Random OS	Alpha = [0, 0.1, 0.4, 0.6, 0.9, 1.0]
20	Naïve Bayes	Known predictors only	SMOTE	Alpha = [0, 0.1, 0.4, 0.6, 0.9, 1.0]
21	Naïve Bayes	Known predictors only	Tomek Links	Alpha = [0, 0.1, 0.4, 0.6, 0.9, 1.0]
22	Logistic Regression	Known predictors with age squared and age-sex interaction	SMOTE	Penalty = None
23	Logistic Regression	Known and BD predictors	SMOTE	Penalty = None
24	Neural Network	BD predictors only	None	Layers = 1<br>Neurons/layer = 1000
25	Neural Network	BD predictors only	None	Layers = 1<br>Neurons/layer = 5000
26	Neural Network	BD predictors only	None	Layers = 2<br>Neurons/layer = 1000
27	Neural Network	BD predictors only	Random US	Layers = 1<br>Neurons/layer = 1000
28	Neural Network	BD predictors only	Random US	Layers = 1<br>Neurons/layer = 5000
29	Neural Network	BD predictors only	Random US	Layers = 2<br>Neurons/layer = 1000
30	Neural Network	BD predictors only	SMOTE	Layers = 1<br>Neurons/layer = 1000
31	Neural Network	BD predictors only	SMOTE	Layers = 1<br>Neurons/layer = 5000

32	Neural Network	BD predictors only	SMOTE	Layers = 2<br>Neurons/layer = 1000
33	Neural Network	BD predictors only	SMOTE	Layers = 3<br>Neurons/layer = 1000
34	Neural Network	BD predictors only	SMOTE	Layers = 4<br>Neurons/layer = 1000
35	Neural Network	Known and BD predictors	None	Layers = 1<br>Neurons/layer = 1000
36	Neural Network	Known and BD predictors	None	Layers = 1<br>Neurons/layer = 5000
37	Neural Network	Known and BD predictors	None	Layers = 2<br>Neurons/layer = 1000
38	Neural Network	Known and BD predictors	Random US	Layers = 1<br>Neurons/layer = 1000
39	Neural Network	Known and BD predictors	Random US	Layers = 1<br>Neurons/layer = 5000
40	Neural Network	Known and BD predictors	Random US	Layers = 2<br>Neurons/layer = 1000
41	Neural Network	Known and BD predictors	SMOTE	Layers = 1<br>Neurons/layer = 1000
42	Neural Network	Known and BD predictors	SMOTE	Layers = 1<br>Neurons/layer = 5000
43	Neural Network	Known and BD predictors	SMOTE	Layers = 2<br>Neurons/layer = 1000

*Where multiple options for hyperparameters are listed in brackets, the best combination was chosen with a grid search.

Resampling methods: synthetic minority oversampling (SMOTE), random undersampling (US), random oversampling (OS)

Neural Network parameters:

Elu activation (except final layer—Sigmoid); He initialization; L2 regularization (Penalty = 0.01); Dropout (20%); Batch normalization; Binary cross-entropy loss; RMS prop optimizer with learning rate 1e-4, rho = 0.9; metrics = AUC; 50 epochs with early stopping
